# Accelerated Wound Closure *In Vitro* by Fibroblasts from a Subgroup of Cleft Lip/Palate Patients: Role of Transforming Growth Factor-α

**DOI:** 10.1371/journal.pone.0111752

**Published:** 2014-10-31

**Authors:** Joël Beyeler, Isabelle Schnyder, Christos Katsaros, Matthias Chiquet

**Affiliations:** 1 Department of Orthodontics and Dentofacial Orthopedics, School of Dental Medicine, University of Bern, Bern, Switzerland; 2 University Clinic for Childrens' Surgery, Bern University Hospital, Bern, Switzerland; National Centre for Scientific Research, ‘Demokritos', Greece

## Abstract

In a fraction of patients surgically treated for cleft lip/palate, excessive scarring disturbs maxillary growth and dento-alveolar development. Since certain genes are involved in craniofacial morphogenesis as well as tissue repair, a primary defect causing cleft lip/palate could lead to altered wound healing. We performed *in vitro* wound healing assays with primary lip fibroblasts from 16 cleft lip/palate patients. Nine foreskin fibroblast strains were included for comparison. Cells were grown to confluency and scratch wounds were applied; wound closure was monitored morphometrically over time. Wound closure rate showed highly significant differences between fibroblast strains. Statistically, fibroblast strains from the 25 individuals could be divided into three migratory groups, namely “fast”, “intermediate”, and “slow”. Most cleft lip/palate fibroblasts were distributed between the “fast” (5 strains) and the “intermediate” group (10 strains). These phenotypes were stable over different cell passages from the same individual. Expression of genes involved in cleft lip/palate and wound repair was determined by quantitative PCR. Transforming growth factor-α mRNA was significantly up-regulated in the “fast” group. 5 ng/ml transforming growth factor-α added to the culture medium increased the wound closure rate of cleft lip/palate strains from the “intermediate” migratory group to the level of the “fast”, but had no effect on the latter group. Conversely, antibody to transforming growth factor-α or a specific inhibitor of its receptor most effectively reduced the wound closure rate of “fast” cleft lip/palate strains. Thus, fibroblasts from a distinct subgroup of cleft lip/palate patients exhibit an increased migration rate into wounds *in vitro*, which is linked to higher transforming growth factor-α expression and attenuated by interfering with its signaling.

## Introduction

Cleft lip and/or palate (CLP) affects overall 1 in 700 live births and represents the most common congenital facial malformation in humans [1]. CLP results from a developmental failure in growth, elevation and/or fusion of the palatal shelves, distinct preformed viscerocranial structures during embryogenesis [2]–[4]. A fraction of patients undergoing primary cleft (especially palatal) surgery show, in comparison to unoperated patients or non-cleft controls, severe disturbances in midfacial growth and dento-alveolar development [5]–[8]. Given many factors involved in treatment outcome, the etiopathogenesis of theses disturbances cannot be adequately attributed to the size of the cleft, the treatment protocol, or the individual growth pattern [9]–[12], but are reportedly due to excessive scarring after primary cleft surgery [9], [13], [14]. In such cases, it is believed that the persistence of tissue remodeling and matrix contracture results in the formation of a rigid collagen-rich tissue [15] that hinders normal maxillary growth [16]. Interestingly, many genes reported to be involved in syndromic and non-syndromic CLP [3] code for transcription factors, growth factors as well as receptors that are known to play key roles in wound repair [4], [17]–[23]. Therefore, one might speculate that a genetic predisposition causing CLP could result in abnormal wound healing subsequent to cleft surgery, and as a consequence influence midfacial growth in a subset of CLP patients. Indeed, children with Van der Woude syndrome (VWS) have an increased risk to wound complications following cleft repair [24]. With 2% of all cases, VWS is the most prevalent syndrome associated with CLP. The syndrome is caused by mutations in *IRF6* [25], a transcription factor involved in the keratinocyte proliferation-differentiation switch [26] and hence in wound repair. In the study cited above [24], 47% of VWS patients developed wound complications after cleft surgery, whereas only 19% of non-VWS children were affected.

By far the largest proportion of CLP cases are nonsyndromic, however [3], and surprisingly, the possibility that a fraction of these might exhibit a clinically relevant susceptibility to abnormal wound healing has not been examined so far. Up to date, mutations and polymorphisms in at least two dozen genes have been associated with nonsyndromic CLP [3], [27], and because of the high number of affected individuals, it seems impractical in the near future to determine the precise genetic cause of the defect for every single patient. Nevertheless, it would be highly desirable to develop a simple method for identifying CLP patients at risk for wound healing problems before they undergo major reconstructive surgery. With this in mind, we asked whether cells isolated from individual CLP patients might exhibit significant differences in their wound healing behavior *in vitro* when compared both relative to each other and to control cells from healthy donors. We thus performed scratch wound assays with cultures of dermal fibroblast strains that were established from lip tissue of 16 CLP patients, excised during their first surgery at 3 months of age. Human foreskin fibroblast strains obtained from 9 children (of which 3 had phimosis) were used as controls. We tested in terms of the rate of cell migration into the wound whether these fibroblast strains were normally distributed or whether they fell into distinct subgroups. Unbiased statistical tests revealed that based on wound closure ability *in vitro*, fibroblasts from the 25 individuals could be divided into three populations, namely “fast”, “intermediate”, and “slow” migratory groups. One third of the CLP fibroblast strains comprised the “fast” migratory group, together with the three phimosis samples. All other CLP strains except one fell into the “intermediate” group. Normal foreskin fibroblast strains were distributed between the “intermediate” and the “slow” group.

In order to identify a possible cause for the observed differences between groups, we next used quantitative RT-PCR to compare the expression levels of various CLP candidate genes among individual fibroblast strains. Interestingly, a significantly higher mRNA expression was found for transforming growth factor alpha (*TGFA)* in “fast” compared to “intermediate” and “slow” migrating strains. Finally, pharmacological studies with agonists and inhibitors of the respective signaling pathway strongly indicated that differences in TGF-α levels are indeed responsible for the distinct migratory behavior of the various CLP fibroblast strains.

TGF-α has been reported to control proliferation, differentiation and carcinogenesis primarily of epithelial cells (for review, see [23]), and to have a role in endochondral ossification [28]. Our present results establish a function for TGF-α in the migration of fibroblasts during wound closure in vitro. Moreover, since *TGFA* is one of the best documented genetic modifiers of facial clefting in humans [29], our current findings might point to a functional link between craniofacial malformation and changed wound healing behavior by dermal fibroblasts in a fraction of CLP patients.

## Materials and Methods

### Ethics statement

This work was performed according to the Ethical Principles for Medical Research Involving Human Subjects as defined by the World Medical Association (Helsinki Declaration). Isolation of human cleft lip (from infants three months of age) and foreskin biopsies (from 2–5 year old boys) for this study has been approved by the Kantonale Ethikkommission Bern, Switzerland (permission number: 170-10). Written consent was obtained from the parents of the children.

### Isolation of human fibroblasts and cell culture

Fresh lip tissue samples, originating from the border between facial skin and oral mucosa, were obtained from CLP patients during surgical closure of the lip at the age of three months. Foreskin tissue samples were obtained from two to five year old boys during routine circumcisions. Tissue was wrapped into sterile cloth wetted with sterile saline, and processed within less than one hour after surgery.

Individual tissue samples (about 0.5 cm^3^ −1.5 cm^3^) were placed in a 10 cm culture dish in 20 ml serum-free Dulbecco's modified Eagle's medium (DMEM; Gibco/Life Technologies, LuBioScience, Lucerne, Switzerland) containing antibiotics/antimycotics (Gibco). They were cut into tiny pieces (<1 mm^3^) with scissors, transferred into a 6 cm culture dish containing 5 ml collagenase D (from Clostridium histolyticum; Roche Diagnostics, Rotkreuz, Switzerland; 1 mg/ml in DMEM), and placed at 37°C in the CO_2_-incubator for 2 hours. Remaining pieces were minced with tweezers for about 15 minutes. 5 ml DMEM containing 10% fetal calf serum (Gibco) was added, and the suspension was triturated for about 10 minutes. After a brief centrifugation at 1100 rpm to remove debris, the supernatant was centrifuged 5 minutes at 2000 rpm. The cell pellet was resuspended in 20 ml 10% FCS/DMEM and transferred into two 10 cm culture dishes (10 ml per dish), which were placed in the CO_2_-incubator. After 48 hours, the medium was changed. After reaching confluency 7 days later, each culture dish was trypsinized and split onto 5 new 10 cm-dishes (in 10 ml 10% FCS/DMEM). The culture media were changed every 3–4 days. Confluent cultures were trypsinized; cells were frozen in 10% dimethyl sulfoxide/20% FCS/DMEM (cells from one dish per vial in 1 ml freezing medium) and stored in the gas phase of a liquid nitrogen tank.

Human primary oral mucosal fibroblasts used for additional comparison were obtained from Dr. Reinhard Gruber (Department of Oral Surgery and Stomatology, University of Bern). These cell strains had been isolated, after approval (Kantonale Ethikkommission Bern) and informed consent, from palatal tissue grafts obtained during gingiva recession coverage in healthy adults as published [30].

All experiments were performed with cells from the first to forth passage. Some strains showed senescent cells starting from the fifth passage; thus later passages were not used for measurements.

### Wounding model in vitro (“scratch wound assay”)

Human fibroblasts were plated into 6-well culture dishes (Cellstar®, Greiner Bio-One, Huberlab AG, Aesch, Switzerland; 700,000 cells/well) in 10% FCS/DMEM and placed at 37°C in the CO_2_-incubator overnight. The next morning, pipet tips were cut obliquely (45° angle) with a sterile razor blade. Wounds ∼1 mm in width were applied by scratching the confluent fibroblast monolayers with the trimmed pipet tips. Cells in suspension were removed by aspiration. Phase contrast images were taken at 0 hours, 5 hours and 24 hours after scratching, using an inverted microscope (Leica DM IL LED) equipped with a 5x/0.12 NA objective and a digital microscope camera (Leica DFC420C, Twain Version 7.7.1.0). Wound closure areas were measured with ImageJ (Software 1.48q, Rayne Rasband, National Institutes of Health, USA) by subtracting the total amount (A) of greyscale pixel counted in the cell-free area remaining after 24 hours from the initial wound area; hence: “Wound closure area” [pixel] = A_initial_ – A_24 hrs_. Since scratch width varied to some extent from one wound to the other, a “relative wound closure” (RWC) area was calculated by normalizing the measured wound closure area (in pixels) to the total area of the image, which covered 2.2×10^6^ pixels (1698×1296 pixels, or 2.04×1.56 mm). Hence: RWC [%] = wound closure area [pixel] × 100 [%]/2.2×10^6^ [pixel], of which 100% is equivalent to 3.18 mm^2^.

### Cell proliferation measurement

Proliferation rates of different CLP strains were determined using a BrdU labeling reagent (Invitrogen, Camarillo, USA) and a biotin-streptavidin based staining kit (Invitrogen, Camarillo, USA). Cells were seeded at 50′000 cells/ml in a 24-well dish and cultured overnight in 10% FCS/DMEM. On the next day, cultures were incubated for 4 hours in sterile-filtered BrdU solution (1∶100 in 10% FCS/DMEM), washed 3×2 minutes with PBS, and fixed with 70% ethanol. Endogenous peroxidase was blocked with 3% H_2_O_2_ for 10 min. BrdU positive nuclei were visualized using biotinylated anti-BrdU, streptavidin-peroxidase and diaminobendizine according to the manufacturer's instructions. Hematoxylin was applied for counterstaining. Images were captured as described above. The ratio of cells in S-phase was determined by the number of BrdU positive cells in relation to the total number of cells per image. Statistical significance was determined by a Student’s t-test. Differences with a value of p<0.05 were considered significant.

### Life imaging and directionality of cell migration

Scratch wounds were applied to fibroblast monolayers as described above. Culture dishes were placed in a live imaging climate chamber (Live Imaging Services, Basel, Switzerland) supplied with 10% CO_2_ and kept at 37°C. Images were captured every 2 minutes during 24 hours with ProgRes CapturePro software by a ProgRes MF_cool_ camera (Jenoptik, Jena, Germany), using a 10x/0.25 NA objective on an Olympus CKX41 inverted microscope. Images were stacked and converted into AVI format using ImageJ software. Directionality was calculated by measuring the euclidean distance (linear distance in pixels between the cell’s starting point at the wound margin and the end position after 24 hours) relative to the accumulated distance (cell path in pixels tracked by line segmentation) using ImageJ. Cell positions were defined by the cell’s nuclei. For one strain of the “fast” and one of the “intermediate” CLP group, directionality of 10 migrating cells was determined. Statistical significance was determined by Student’s t-test. Differences with a value of p<0.05 were considered significant.

### Pharmacological studies

To determine the optimal dose for each growth factor or inhibitor, preliminary scratch wound assays were performed with various concentrations using at least one fibroblast strain of each CLP migratory group (“fast” and “intermediate”) before proceeding with other cell strains. The concentrations were based on the technical data provided by the manufacturers: 2, 5, and 20 ng/ml for recombinant human TGF-α (SIGMA-Aldrich, Buchs, Switzerland); 0.25, 0.5, and 2.5 µg/ml for neutralizing TGF-α antibody (BioVision, Milpitas, USA); 5, 20, and 50 ng/ml for recombinant human PDGF-CC (SIGMA-Aldrich, Buchs, Switzerland). PD153035 (Stelleckchem, Houston, USA) was examined at 150 nM, 600 nM and 1 µM, and Lapatinib (Stelleckchem, Houston, USA) at 150 nM, 600 nM, 2 µM, and 5 µM. Stock solutions of PD153035 and Lapatinib were prepared in DMSO and diluted in serum-free DMEM before addition to the culture media. Confluent cultures were pre-incubated with all reagents 3 hours before scratch wounds were applied. Each reagent was tested on at least three randomly chosen cell strains per group. The experiments were repeated twice.

### Immunofluorescence staining

Wounded cell monolayers were fixed in 4% paraformaldehyde diluted in PBS (150 mM NaCl, 20 mM Na phosphate, pH 7.4), then blocked and permeabilized with 3% BSA/0.2% TritonX-100/PBS for 30 minutes at room temperature. Cell cultures were incubated for 45 minutes with mouse monoclonal anti-vinculin antibody (SIGMA-Aldrich, Buchs, Switzerland) diluted 1∶1000 in 3% BSA/0.2% Triton/PBS. Prior to adding the secondary antibody, samples were washed three times for 5 minutes with 0.5% BSA/0.03% TritonX-100/PBS. TRITC-phalloidin (1 µg/ml; SIGMA-Aldrich, Buchs, Switzerland) was added along with secondary antibody Alexa Fluor® 488 goat anti-mouse IgG (Invitrogen, Life Technologies, LuBioScience, Lucerne, Switzerland; diluted 1∶1000 in BSA/Triton/PBS). Cells were washed three times for 5 minutes with BSA/Triton/PBS and mounted in PBS-buffered 90% glycerol containing DAPI (Roche, Basel, Switzerland) at a concentration of 1 µg/ml. Images were captured with ProgRes CapturePro software by a ProgRes CT3 camera (Jenoptik, Jena, Germany), using a 40x/0.75 NA objective on an Olympus BX-51 phase/fluorescence microscope equipped with a xenon lamp (X-Cite, series 120PC Q, Ontario, Canada), and fluorescence filters U-MWIBA3 for AlexaFluor 488, U-MWIGA3 for Alexa Fluor 568 and TRITC, and U-MNUA2 for DAPI.

The size and shape of focal adhesions from “fast” and “intermediate” CLP fibroblast strains was measured from vinculin immunofluorescence micrographs by ImageJ. Images were thresholded, and areas of interest corresponding to the front ends of individual cells were selected manually. Thresholded particles (i.e. vinculin-positive focal adhesions) were fitted with ellipses, and their area, length, and aspect ratio was calculated individually. Of each fibroblast strain, 250–400 focal adhesions from 12–15 cells were measured and data averaged.

### RNA extraction and cDNA synthesis

Human fibroblasts of the second passage were plated on a culture dish in 10% FCS/DMEM and placed at 37°C in the CO_2_-incubator. At 90% confluency, cells were washed with PBS. Total RNA was isolated using the innuPREP RNA Mini Kit (Analytik Jena AG; LifeScience, Jena, Germany). The extracted RNA was dissolved in 80 µl of RNase-free distilled water and stored at −80°C until use. RNA quantification was performed using Nanodrop 2000c (Thermo Scientific, Waltham, MA, USA).

For cDNA synthesis, 500 ng RNA in 14 µl ddH_2_O was tagged with 1 µl Oligo(dT) 15 Primer (0.5 mg/ml; Promega, Dübendorf, Switzerland) at 70°C during 5 minutes. Samples were placed for 5 minutes on ice prior to adding 5 µl M-MLV 5xbuffer (Promega, Dübendorf, Switzerland), 1.25 µl dNTPS (10 mM; Promega, Dübendorf, Switzerland), 3.25 µl ddH_2_O and 0.5 µl M-MLV reverse transcriptase (Promega, Dübendorf, Switzerland). The final solution was heated for 60 minutes at 40°C, followed by 15 minutes at 70°C to stop the reaction. Samples were placed for 5 minutes on ice, centrifuged and stored at −80°C. RNA extraction was performed with all cell strains of the “fast” and “slow” migratory groups, whereas for the “intermediate” group, RNA isolation was limited to six strains (2 Fsk, 4 CLP).

### Design of primers

All primers were individually designed and tested for their specificity using the online Gene Bank database and the Primer-BLAST software provided by the National Center for Biotechnology Information for human *TGFB1, TGFB3, BMP7, FGF12, EGF, TGFA, PDGFC, TGFBR2, FGFR1, EGFR, PDGFRB, MET, JAG1, TNC, TNW, FN, COL1, COL3, MMP2, MMP9, VCL, ACTA2, ADH1C, IRF6, RUNX2, SOX9* and *GAPDH* (see Table S1). In the center of each probe sequence, an exon-exon junction was placed to avoid non-specific fluorescent emission derived from contaminating genomic DNA. The nucleotide sequences and size of the primers are shown in Table S1. The designed primers were purchased from Microsynth (Balgach, Switzerland), diluted in ddH_2_O (10 µM) and stored at −20°C.

### Real-time quantitative RT-PCR

Real-time quantitative RT-PCR was performed using 20 µl total reaction volume containing 1xPowerSYBR® green (LuBioScience, Lucerne, Switzerland), 0.5 µM forward/reverse primers and 20 ng cDNA in ddH_2_O on a 7500 Real-Time PCR System (Applied Biosystems, Carlsbad, CA, USA). Reactions were performed in duplicates and repeated at least two times. Data were analyzed by the ΔCt method [31]. Each Ct value was normalized against that of GAPDH in the same reaction. After averaging all ΔCt values for each target gene for an individual cell strain, the relative mRNA expression (2^−ΔCt^) was calculated.

### Statistics

All statistical tests and graphs were performed and designed using the software R (version 2.15.1). For each experiment, the data were first tested for normality (Shapiro-Wilk test) and homoscedasticity (Levene’s test). As a result to these tests, a Kruskal-Wallis test was run followed by a pairwise Wilcoxon rank sum test with p-value adjustment [32] for evaluating the significance of differences between more than two strains (n [wounds per strain] = 38) or migratory groups (k [strains per group] ≥4). An unpaired two-sample Wilcoxon test was conducted to test the significance between two sub−/groups. Differences with a value of p<0.05 were considered significant (*p<0.05, **p<0.01, ***p<0.001). Boxplots of individual strains include the averages of at least three independent measurements, whereas boxplots that represent either population groups or subgroups include the average per individual cell strain. Whiskers indicate the maximum and minimum values measured.

## Results

### Human fibroblast strains of various origin exhibit significant differences in their rate of wound closure *in vitro*

Wound healing assays were performed *in vitro* with primary human fibroblasts isolated from excess lip tissue of 16 cleft lip/palate (CLP) patients, and with human foreskin fibroblasts derived from 6 healthy boys (Fsk) and 3 patients with phimosis (Phim). All these cell strains were obtained from infants (see Materials and Methods). Note that lip and foreskin fibroblasts are comparable in their tissue origin since they are both derived from a mucocutaneous zone (a border between epidermis and mucosa). Scratch wounds were inflicted to confluent fibroblast monolayers on tissue culture dishes. Wound areas were measured morphometrically at time zero and after 24 hours, and for each fibroblast strain the relative wound closure area (RWC; expressed as % of the total area of the image, see Materials and Methods) was determined from the average of at least three independent experiments, which were performed with different passages from individual strains. A Kruskal-Wallis test followed by an unbiased pairwise Wilcoxon rank sum test with Benjamini & Yekutieli [32] correction for multiple comparisons was then performed on the entire data set, in order to determine whether the velocity of wound closure (corresponding to the rate of cell migration into the wound) was distributed normally between fibroblast strains, or whether they fell into distinct populations. Indeed, fibroblasts derived from the 25 individuals of our cohort migrated with statistically distinct speeds into the wounds inflicted *in vitro*; the mean ranks of the RWC per cell strain were significantly different among the 25 individual strains (Kruskal-Wallis chi-squared = 541.8508, df = 24, p<2.2×10^−16^; n = 38 per strain). Shapiro-Wilk normality test (p<0.001), and Levene’s test (p>0.08) indicated that the assumptions of Kruskal-Wallis test were respected.

**Figure 1:**
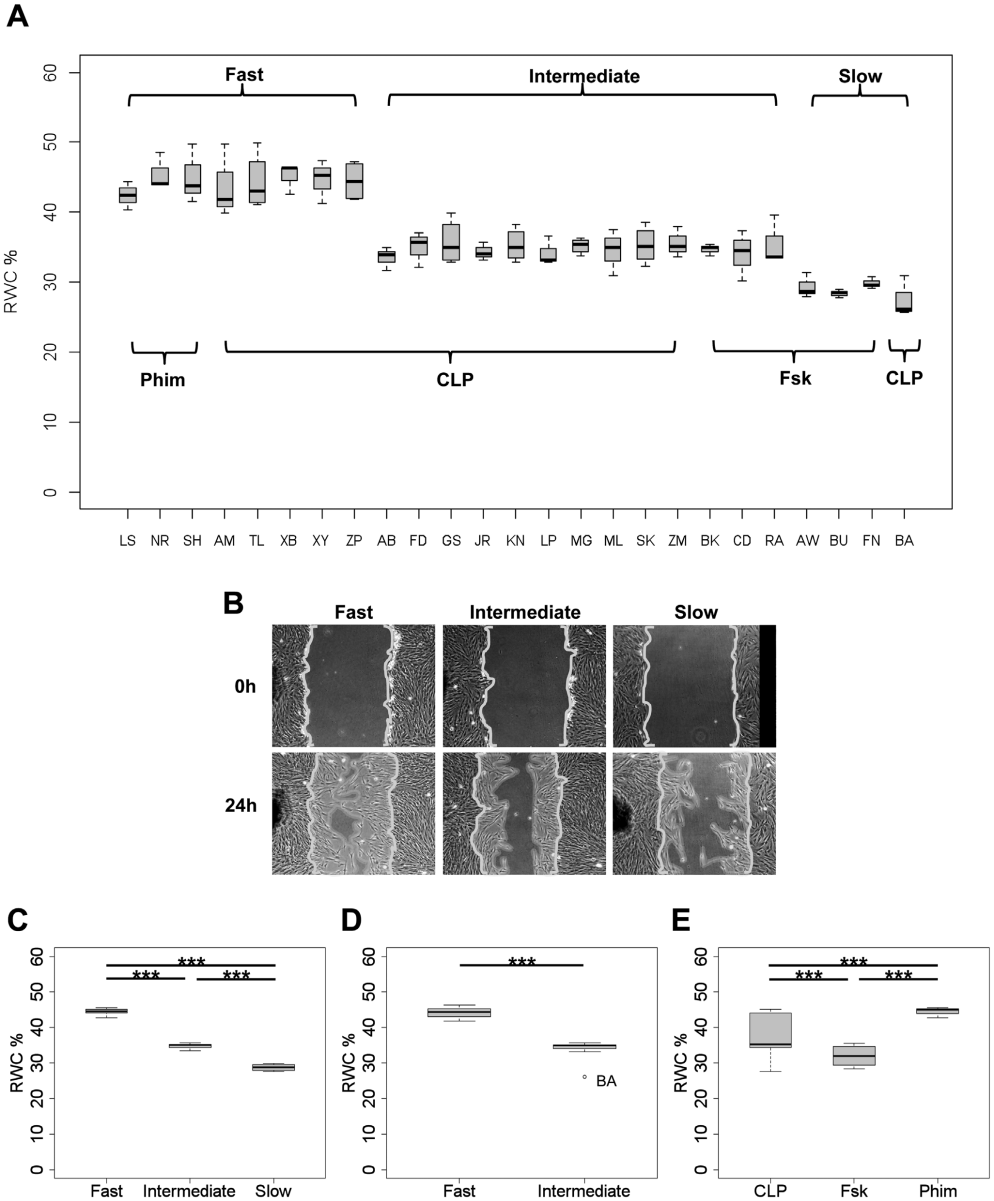
Fibroblasts from CLP patients fall into statistically distinct groups based on wound closure *in vitro*. Fibroblast strains were obtained from 16 CLP patients, 6 healthy individuals (foreskin; Fsk) and 3 patients with phimosis (Phim), and scratch wound assays were performed *in vitro* (see Materials and Methods). Box plots show the percentage of relative wound closure after 24 hours (% RWC) and the distribution of the collected data of at least three independent experiments (***p<0.001). (A) Box plots of the individual cell strains are marked at the bottom of the graph by the initials of the patients; they cluster into three distinct migratory groups namely “fast”, “intermediate” and “slow”. The origin of strains from the different proband groups is also indicated: Phimosis (Phim), cleft lip/and palate (CLP), normal foreskin (Fsk). See Tables S2 and S3 for descriptive data and p-values, respectively. (B) Representative images of one strain from each of the three migratory groups, taken immediately after wounding (0 h) and 24 hours later (24 h). *Area of scale bar corresponds to 10% RWC or 0.32* *mm^2^*. (C) Box plots of the median RWC values obtained for all cell strains within each of the three migratory groups. (D) Two distinct migratory groups within the CLP patient cohort are evident after performing a multiple comparisons test with the data collected from CLP fibroblast strains alone. The fibroblast strain derived from patient “BA” was considered an outlier and therefore not included in this statistical analysis. See Tables S2 and S4 for the descriptive data and p-values, respectively. (E) When CLP, Fsk and Phim cell strains were grouped separately, they corresponded to statistically distinct populations, although heterogeneity within the CLP group was evident from the large variance.

Depending on the RWC, fibroblast strains could be divided into three migratory groups, namely “fast”, “intermediate”, and “slow” (Fig. 1A, B; Table S2), which were significantly different from one another. The p-values after multiple comparisons for the individual strains are listed in Table S3. The “fast” group included the 3 phimosis and 5 of the CLP strains. Their median RWC ranged between 41.8% and 46.4%. In the intermediate group, for which the RWC varied from 33.2% to 35.8%, 10 CLP and 3 normal foreskin strains were categorized. The slowest and smallest group (RWC = 26.1% –29.6%) was represented by 3 normal foreskin strains and 1 CLP “outlier” (strain “BA”). The migratory phenotype of each fibroblast strain within a group was stable when assays were repeated with different passages of cells from the same individual, as evidenced by the small variance of the combined measurements for individual strains (Fig. 1A). Statistically, these results were also confirmed by one-way ANOVA (p<2.2×10^−16^) followed by Tukey’s posthoc test using the means of each strain (“fast”, n = 8; “intermediate”, n = 13; “slow”, n = 4) for each migratory group (Fig. 1C; Table S2).

### Fibroblasts derived from cleft lip/palate patients fall into two distinct groups based on wound closure rate *in vitro*

A major aim of our study was to compare the cohort of CLP fibroblast strains not only with healthy controls, but also among each other, in order to see whether their migration rates were normally distributed or rather fell into distinct subclasses. Indeed, within the CLP cohort the mean ranks of the RWCs per cell strain were significantly different (Kruskal-Wallis chi-squared = 261.3335, df = 14, p-value <2.2×10^−16^). Two distinct CLP migratory sub-groups, “fast” and “intermediate”, were evident (Fig. 1D; Table S2) after conducting a pairwise Wilcoxon rank sum test with Benjamini & Yekutieli [32] correction (see Table S4). With a ratio of 5 out of 16, about one third of the CLP strains had significantly greater RWCs than the remainder. The median RWC was 44.2% for the “fast” CLP migratory group and 34.8% for the “intermediate” group. The only “slow” CLP strain “BA” was considered an outlier when assigned to the intermediate group, and hence was not included in statistical analysis of the CLP cohort.

Kruskal-Wallis test followed by a pairwise Wilcoxon rank sum test also indicated that when data were grouped according to CLP, Fsk and Phim, respectively, these subject groups represented statistically distinct populations (Kruskal-Wallis chi-squared = 27.2245, df = 2, p-value = 1.2×10^−06^) (Fig. 1E; Table S2). Phim was the subject group with the highest median RWC (44%). Fsk had the lowest RWC (29.0%), which was significantly below the median RWC of Phim (p<3×10^−06^). The differences between CLP (median RWC 37.1%) and both, Fsk and Phim groups were also significant (p<0.001 and p<0.003, respectively). However, one-way ANOVA, using the means of each strain within the subject groups (CLP, k[strains] = 16; Fsk, k = 6; Phim, k = 3), followed by Tukey’s posthoc test, revealed significant differences between Fsk and Phim (p<0.003), but not between CLP and both Fsk and Phim groups (p>0.05). These results from ANOVA were a further indication for heterogeneity within the CLP group. To confirm this notion, the two CLP groups specified above were compared separately to normal foreskin fibroblasts (not shown in graph). Indeed, the RWC values of the “fast” CLP group were significantly different from those of the Fsk group (p = 0.012), but not of the “intermediate” group (p = 0.167).

In summary, our results indicate that the majority of CLP fibroblast strains (the “intermediate” group) migrates into wounds *in vitro* at a similar velocity as normal infant foreskin fibroblasts, whereas a minority of CLP strains (the “fast” group) exhibit significantly enhanced migration rates.

Since donor age and tissue origin might affect the wound healing rate *in vitro*, as an additional control we performed scratch assays with oral mucosal (palatal) fibroblasts obtained from three normal adult individuals. We found that one of the three strains fell into the “intermediate” and two into the “slow” migratory group (Fig. S2). In any case, none of the normal adult oral strains migrated at an increased rate like “fast” CLP lip fibroblasts, providing additional evidence for the exceptional properties of the latter.

### Enhanced wound closure by “fast” CLP fibroblasts is due to increased migration velocity

The difference in the speed of wound closure *in vitro* between “fast” and “intermediate” CLP fibroblast strains was not caused by different rates of cell proliferation, since the proportion of cells in S-phase during a 4 hour period of labeling with BrdU was essentially the same for both groups (Figure S3). Direct counting of number of mitoses per 24 hours from life movies of scratch assays confirmed this result (not shown). Life imaging (see Movies S1 and S2) further revealed that the average migration distance per cell per 24 h in the wound area was almost twice as high for fibroblasts from a “fast” compared to an “intermediate” CLP strain (602±258 versus 324±114 µm; p<0.01), whereas the directionality of cell movement was not significantly different (“fast”: 0.55±0.22; “intermediate”: 0.71±0.20). Thus, these results show that the enhanced speed of wound closure by the “fast” strains is due to increased velocity of cell migration, but not to increased proliferation rate or directionality of movement.

### Focal adhesion size of migrating fibroblasts differs between “fast” and “intermediate” CLP groups

**Figure 2:**
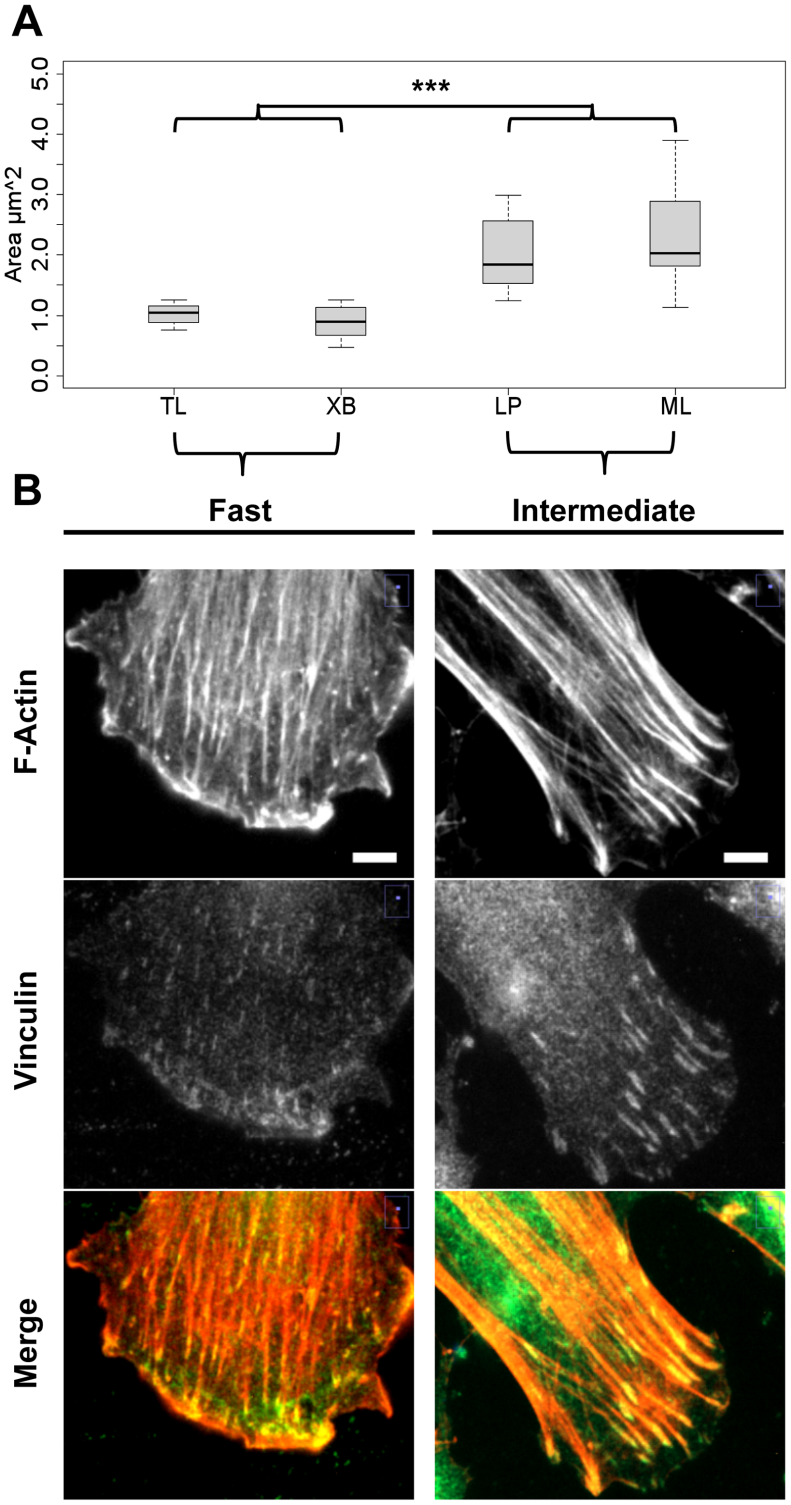
Size of focal adhesions differs between the two CLP migratory groups. Immunofluorescence staining against vinculin, and rhodamine-phalloidin staining for F-actin of migrating cells 24 h after scratch wounds were applied. Two randomly chosen strains from the “fast” (TL and XB) and the “intermediate” (LP and ML) group, respectively, were included in the analysis. (A) The box plots indicate the area in µm^2^ covered by individual vinculin positive focal adhesions at the front end of cells (n[cells per strain] = 12–15). The whiskers indicate the maximum and minimum area per focal adhesion in a strain (***p<0.001). (B) Representative pictures from two strains depict the front end with the lamellipodium of individual cells migrating into the wound. Typically, migrating cells of the “fast” CLP group form smaller focal adhesions in comparison to cells of the “intermediate” group. *Scale bar, 10 µm.*

Immunofluorescence staining for vinculin and F-actin was conducted on wounded cultures to examine whether focal adhesions of migrating fibroblasts at the wound edge differed between “fast” and “intermediate” CLP strains. For this experiment, two representative strains of each CLP group were randomly chosen, stained, and evaluated. Vinculin positive focal adhesions at and behind the front lamellipodium of individual migrating cells were measured; we quantified the area (in µm^2^) covered by single adhesion contacts. As shown in Fig. 2A, the average size of focal adhesions was significantly smaller in fibroblasts from “fast” migrating strains compared to strains of the “intermediate” CLP group (p<7×10^−09^). Images showed in addition that cells from “intermediate” strains tended to have fewer but thicker stress fibers attached to their large focal adhesions, whereas “fast” migrating fibroblasts had prominent lamellipodia, and showed thinner and more evenly distributed actin fibers connected to many small adhesion contacts (Fig. 2B; Fig. S1). As expected, these observations indicate that focal adhesion size and actin organization correlate with migratory behavior and thus with wound closure ability of the respective CLP strains in wounding assays.

### Increased wound healing in vitro correlates with changed expression of *TGFA* and *PDGFC*

**Figure 3:**
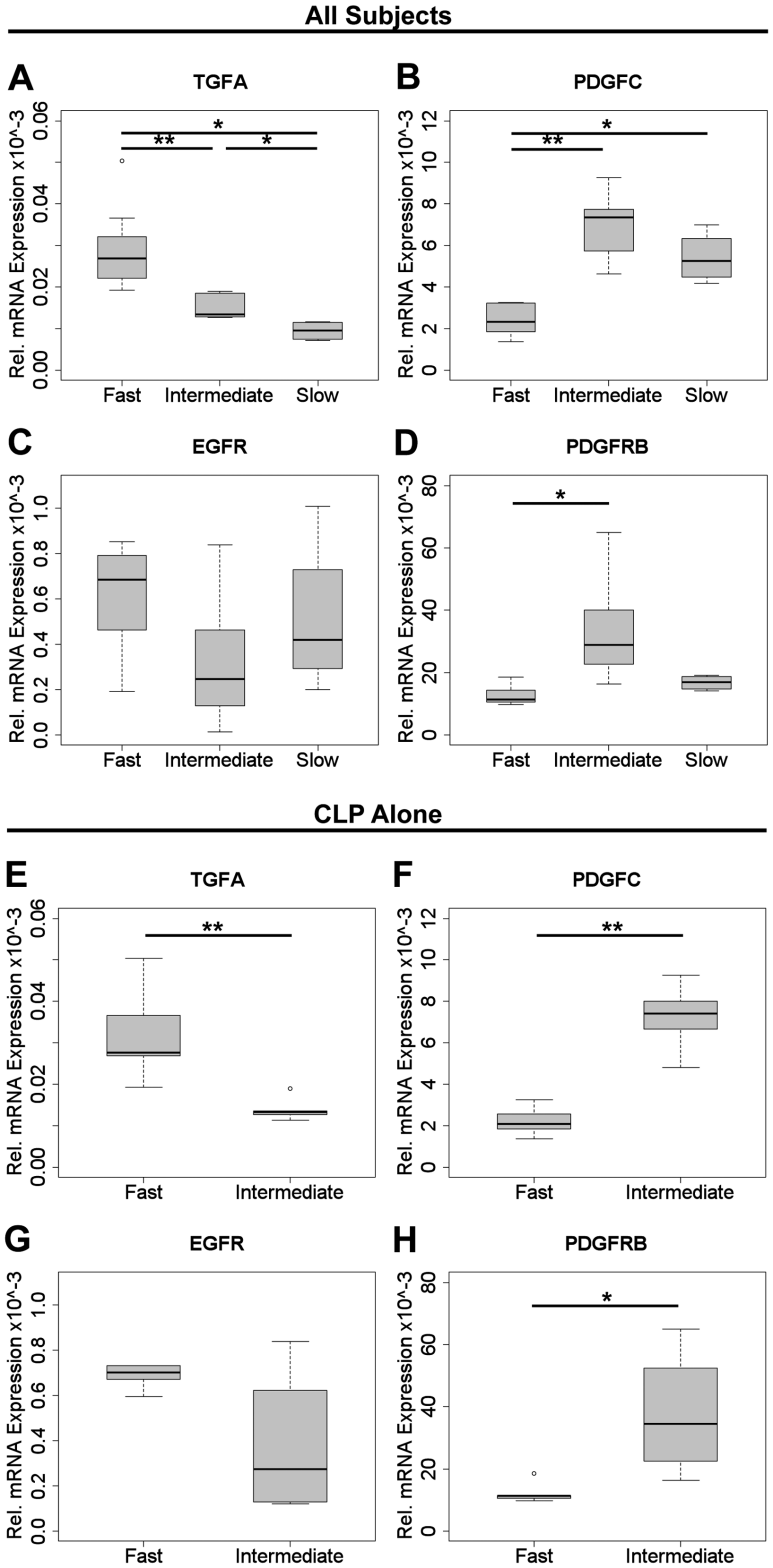
Different expression levels of *TGFA, PDGFC, EGFR* and *PDGFRB* in the three migratory groups. Expression levels were determined for the various mRNAs by qRT-PCR and normalized relative to GAPDH. Results are represented by boxplots; whiskers indicate the maximum and the minimum values in each group (*p<0.05; **p<0.01). (A) *TGFA* was significantly up-regulated in the “fast” migratory group. The difference between the “intermediate” and “slow” migratory groups was also significant. (B) *PDGFC* was down-regulated in the “fast” migratory group compared to “slow” and “intermediate” migratory groups. The difference between the “slow” and “intermediate” migratory group was not significant. Expressions levels of the respective receptors *EGFR* and *PDGFRB*: (C) The mRNA of *EGFR* showed roughly the same tendency, but was not significant. (D) *PDGFRB* was significantly down-regulated in “fast” migratory group compared to the “intermediate” migratory group. Differences in expression level of the same mRNAs were persistent when comparing the “fast” with the “intermediate” migratory group within the CLP cohort: (E) *TGFA*; (F) *PDGFC*; (G) *EGFR,* and (H) *PDGFRB*.

We next asked whether and which genes known to be involved in both facial morphogenesis and regeneration might be responsible for the differences observed between fibroblast strains in their speed of wound closure *in vitro*. Therefore, fibroblasts from the second passage of individual strains were grown to near confluency and their RNA was isolated. The mRNA expression of *TGFB1, TGFB3, BMP7, FGF12, EGF, TGFA, PDGFC, TGFBR2, FGFR1, EGFR, PDGFRB, MET, JAG1, TNC, TNW, FN, COL1, COL3, MMP2, MMP9, VCL, ACTA2, ADH1C, IRF6, RUNX2, SOX9* was measured by qRT-PCR, normalized against *GAPDH*, and statistically evaluated by Kruskal-Wallis followed by a pairwise Wilcoxon rank sum test for multiple comparisons. When comparing cell strains derived from the entire cohort (CLP, Fsk, and Phim subject groups), we did not find significant differences in expression level for most of these genes (not shown). However, our results indicated that the mean rank of *TGFA* mRNA expression was significantly higher (>2-fold; Fig. 3A) and of *PDGFC* lower (>3-fold; Fig. 3B) in the “fast” migratory group compared to the “intermediate” and “slow” groups. Interestingly, genes coding for respective receptors of these growth factors showed the same tendency, although differences were only significant for *PDGFRB* (>2-fold, Fig. 3D), not for TGF-α receptor *EGFR* (Fig. 3C).

When fibroblast strains from the CLP patient cohort were evaluated separately, the difference in expression between “fast” and “intermediate” migratory groups was again significant for both *TGFA* (>2-fold; Fig. 3E) and *PDGFC* (>3-fold; Fig. 3F). The corresponding receptors *EGFR* (Fig. 3G) and *PDGFRB* (>2-fold, Fig. 3H) exhibited the same trend.

### Addition of exogenous TGF-α accelerates wound closure by fibroblasts primarily from the “intermediate” migratory CLP group

**Figure 4:**
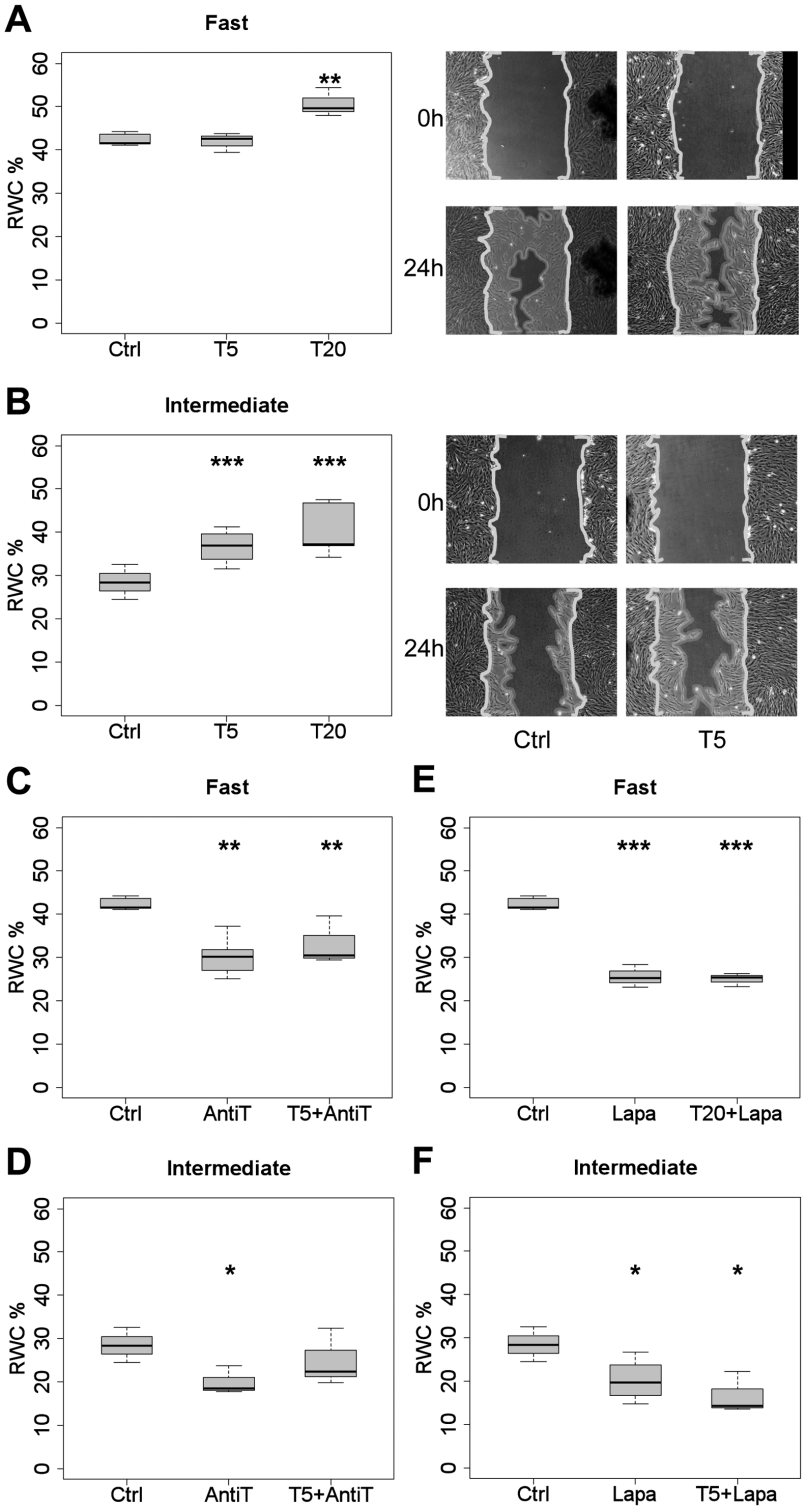
Effect of TGF-α, anti-TGF-α, and EGFR/ERBB2-inhibitor on wound closure by “fast” versus “intermediate” CLP strains. Boxplots depict the RWC in scratch wound assays at 24 h of “fast” (A, C, E) and “intermediate” (B, D, F) CLP strains, in either the absence (control; Ctrl) or the presence of the following agents diluted in 10% FCS/D-MEM: TGF-α at 5 ng/ml (T5) or 20 ng/ml (T20); TGF-α neutralizing antibody at 0.5 µg/ml (AntiT); Lapatinib at 5 µM (Lapa); TGF-α plus anti-TGF-α (T5+AntiT); or TGF-α plus Lapatinib (T5+Lapa; T20+Lapa) (*p<0.05, **p<0.01, ***p<0.001). The micrographs show representative examples of scratch wound assays at 0 and 24 h in the absence or presence of the drugs indicated at the bottom. *Area of scale bar corresponds to 10% RWC or 0.32* *mm^2^*.

In a first attempt to find out whether the observed differences in TGF-α expression levels between “fast” and “intermediate” CLP groups might be causally linked to the speed of wound closure by individual fibroblast strains, we tested the effect of adding exogenous growth factor to the cultures during the assay. Three hours before wounds were inflicted to fibroblast monolayers, TGF-α was added to the standard culture media at either 5 ng/ml or 20 ng/ml. The addition of 5 ng/ml TGF-α significantly increased the RWC (∼1.5-fold; p<7×10^−05^) in the “intermediate” CLP group, whereas it had no effect in the “fast” group (Fig. 4A, B). The higher concentration of TGF-α (20 ng/ml) caused an increase in both groups, but less in the “fast” (∼1.2-fold, p<7×10^−03^) than in the “intermediate” (∼1.4-fold, p<1×10^−05^) (Fig. 4A, B).

### TGF-α neutralizing antibody decreases wound closure by CLP fibroblasts

Since supplementing the culture media with exogenous TGF-α stimulated wound closure, we asked whether the contrary effect was achieved by administering.

TGF-α-neutralizing antibody to the culture media. A significant decrease of the RWC was observed with 0.5 µg/ml anti-TGF-α in both “fast” (∼0.7-fold, p<3×10^−03^) and “intermediate” (∼0.7-fold, p<0.05) groups (Fig. 4C, D). A concentration of 0.25 µg/ml anti-TGF-α did not cause a significant decrease of the RWC in all strains, whereas 2.5 µg/ml was no more efficient than 0.5 µg/ml in inhibiting wound closure (not shown). To confirm that the antibody indeed blocked TGF-α activity, we also tested combinations of TGF-α with anti-TGF-α. As we expected for the “intermediate” group, the stimulating effect of 5 ng/ml TGF-α was abrogated when 0.5 µg/ml TGF-α neutralizing antibody was added in addition to the culture media (Fig. 4D). In the “fast” group, the RWC was reduced by TGF-α plus anti-TGF-α to the same level as by anti-TGF-α alone, indicating that the antibody was able to block both endogenous and exogenously added TGF-α (Fig. 4C).

### Inhibiting EGFR/ERBB2 signaling decreases wound closure by CLP fibroblasts

To study the role of TGF-α in wound closure further, we tested whether inhibiting the signaling by its receptor EGFR would reduce wound closure ability in both CLP groups. To this aim, first attempts were conducted with the specific EGFR tyrosine phosphorylation inhibitor drug PD153035. For the “fast” group, the reduction in RWC was rather small but significant (∼0.9-fold, p<1×10^−04^) at 600 nM in 10% FCS/DMEM. No effect was measured with 600 nM or 1 µM PD153035 in the “intermediate” group (data not shown).

The EGF receptor (ERBB1) can either form homodimers or heterodimerize with other members of the EGFR/ERBB superfamily, such as ERBB2 [33]. With this in mind, we tested an EGFR/ERBB2 tyrosine phosphorylation inhibitor, namely Lapatinib. A significant decrease of the RWC was measured with 5 µM of this inhibitor for both groups relative to the controls (“fast” ∼0.6-fold, p<8×10^−06^; “intermediate” ∼0.7-fold, p<0.05) (Fig. 4E, F). Further, we examined whether the effect of Lapatinib persisted in presence of exogenously added TGF-α: Wounding assays were performed after preincubating cultures with 5 or 20 ng/ml TGF-α in combination with 5 µM Lapatinib. The relative wound closure was significantly decreased compared to the controls in both groups, to approximatively the same level as with Lapatinib alone (Fig. 4E, F). Interestingly, “fast” migratory fibroblast strains were reduced to about the RWC of the “intermediate” group after administration of Lapatinib to the culture media. This confirms again the contribution of the TGF-α/EGFR signaling pathway to the existence of distinct CLP migratory groups.

### “Intermediate” CLP group is stimulated by TGF-α under low-serum conditions

**Figure 5:**
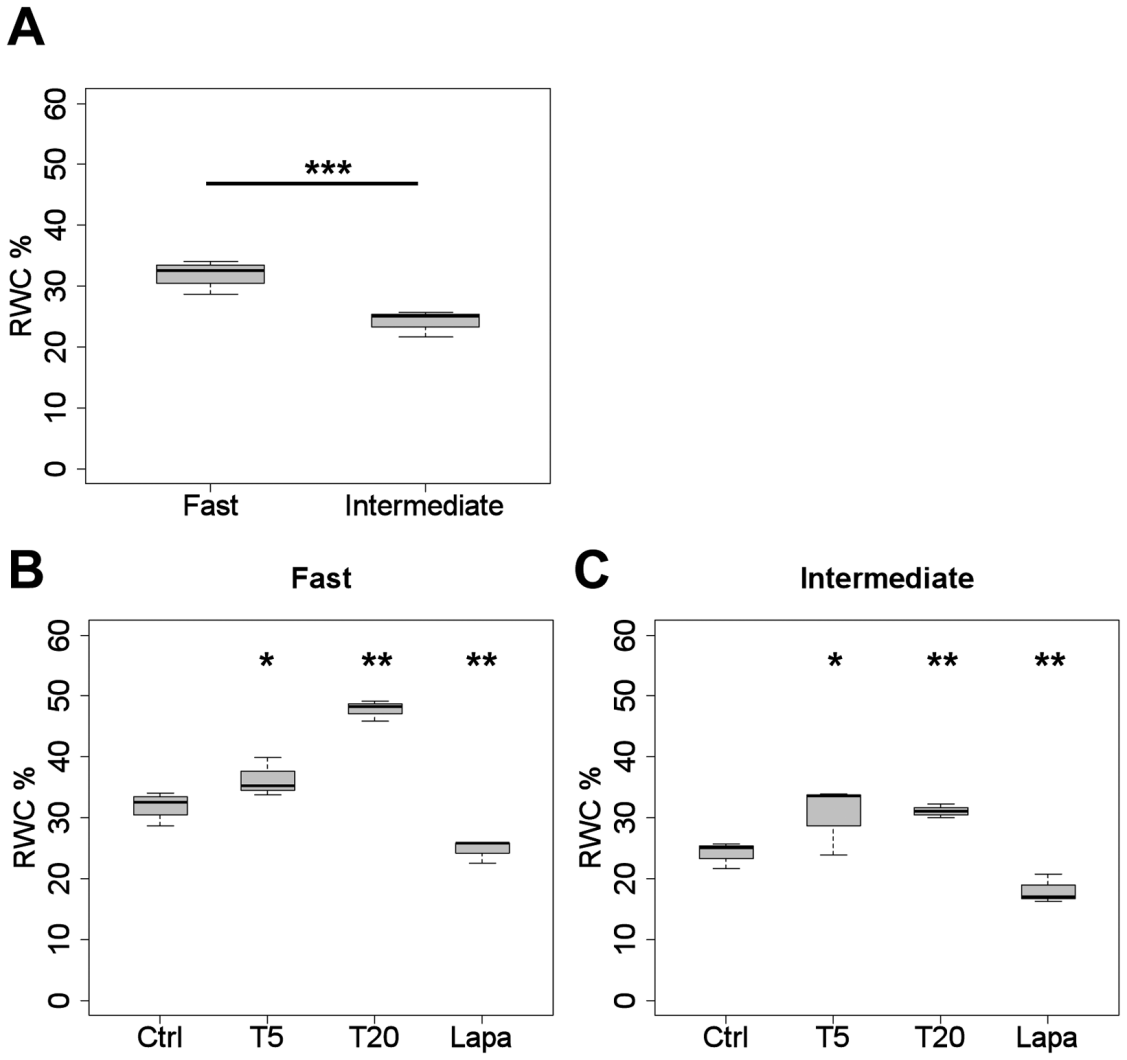
Distinct CLP migratory groups persist under low serum conditions. The graphs show the RWC in scratch wound assays at 24 h in 0.3% FCS/DMEM (*p<0.05, **p<0.01, ***p<0.001). (A) Despite a general decrease of the RWC, “fast” and “intermediate” CLP groups could still be distinguished under low-serum conditions. (B, C) Effect of exogenous TGF-α diluted at 5 ng/ml (T5) and 20 ng/ml (T20), as well as of 5 µM Lapatinib (Lapa) on both CLP subgroups under low serum conditions in comparison to controls (Ctrl).

All experiments with TGF-α and inhibitors described so far were done in standard medium containing 10% serum, which simulates conditions in a wound *in vivo*. Since serum contains other growth factors (such as platelet-derived PDGF-BB) that might independently or synergistically stimulate fibroblast migration in scratch wound assays, it was important to test whether effects of TGF-α were persistent in low-serum conditions. Scratch wound assays were conducted with CLP strains in 0.3% FCS/DMEM culture media. Also in low serum, the difference in the speed of wound closure between “fast” and “intermediate” CLP groups was significant (p<4×10^−05^): The RWC of the “fast” group was ∼1.3-fold higher than the one of the “intermediate” group (Fig. 5A). Next we tested the wound closure ability after addition of either TGF-α or Lapatinib to both groups. The “intermediate” group was rescued by 5 ng/ml TGF-α in terms of the RWC (∼1.3-fold, p<0.02; Fig. 5C). A significant but rather small increase was measured for the “fast” group (∼1.1-fold, p<0.02; Fig. 5B). The higher concentration of TGF-α caused an increase in both groups (“fast” ∼1.5-fold, p<2×10^−03^, “intermediate” ∼1.2-fold, p<0.04; Fig. 5B, C). When lapatinib was preincubated with cultures at 5 µM in low-serum media, the RWC of both the “fast” and the “intermediate” group was significantly decreased (p<0.01) (Fig. 5B, C). Thus, the effects of TGF-α or 5 µM Lapatinib seen under high-serum conditions could in essence been reproduced in medium containing only 0.3% FCS, although the RWCs were generally lower.

### Small effects of PDGF-CC on wound closure by CLP fibroblasts

**Figure 6:**
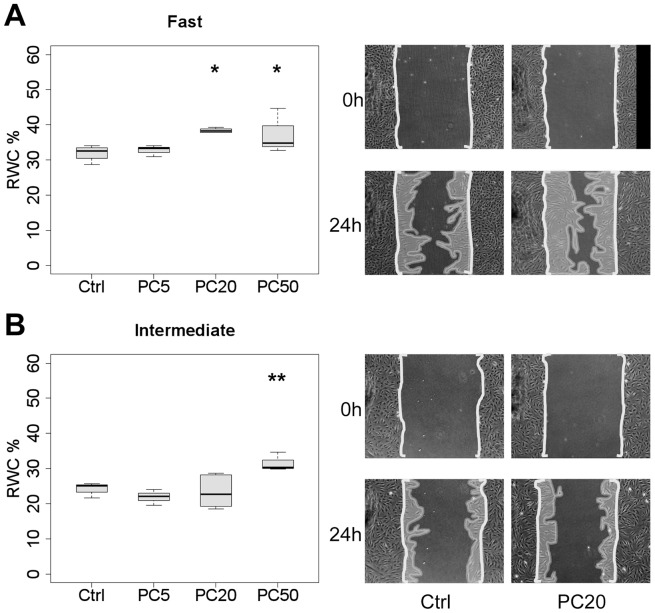
Small effect of PDGF-CC on wound closure by fibroblasts *in vitro*. Boxplots represent the RWC in scratch wound assays at 24 h (*p<0.05, **p<0.01) of “fast” (A) and “intermediate” (B) CLP strains in 0.3% FCS/DMEM in the absence (control; Ctrl) or the presence of PDGF-CC diluted at either 5 ng/ml (PC5), 20 ng/ml (PC20) or 50 ng/ml (PC50). Micrographs show representative examples at 0 and 24 h in the absence (Ctrl) or presence of PDGF-CC (P20; P50). *Area of scale bar corresponds to 10% RWC or 0.32* *mm^2^*.

As we have shown in Fig. 3, *PDGFC* and *PDGFRB* were also differentially expressed between migratory groups, suggesting a possible contribution of cellular PDGF-CC to wound closure in scratch assays. Since *PDGFC* was more highly expressed in the “intermediate” group, however, we expected little effect of exogenously added PDGF-CC in terms of changes in the RWC. To minimize a possible contribution of PDGF-BB from serum, the following experiments were conducted in low-serum conditions. PDGF-CC was added to cultures at 5, 20, and 50 ng/ml in 0.3% FCS/DMEM. For the “intermediate” group, the highest concentration somewhat increased the speed of wound closure (∼1.2-fold, p<8×10^−03^; Fig. 6B); lower concentrations in the physiological range did not have any effect. For the “fast” group, small increases of the RWC were measured at 20 ng/ml (∼1.2-fold, p<0.02) and 50 ng/ml (∼1.1-fold, p<0.03), but not at 5 ng/ml. In summary, high concentrations of PDGF-CC appeared to slightly stimulate the speed of wound closure for both “intermediate” and “fast” migratory strains, but effects were small compared to TGF-α even at 5 ng/ml.

## Discussion

Fibroblasts are the most important cells in wound contraction, granulation tissue formation and scarring [34]. During migration into the wound, fibroblasts generate tractional forces, thus transmitting mechanical stress to the extracellular matrix [35], [36]. Both, mechanical stress and TGF-β1 promote differentiation of fibroblasts to myofibroblasts (reviewed in [37]). Mature myofibroblasts synthesize extracellular matrix and contribute to wound contracture, and thereby increase tension development in a positive feedback loop [37]. Therefore, the initial extent of fibroblast migration into the wound contributes to the later differentiation of myofibroblasts and favors fibrogenesis. The simple *in vitro* scratch wound assay used here has been shown to reveal differences in migratory behavior between fibroblasts from various sources that correlate with aberrant wound healing *in vivo*. For example, fibroblasts isolated from keloid tissue were reported to have significantly higher migration rates in scratch wound assays than extralesional fibroblasts from the same individual [38].

Using this *in vitro* wound healing assay, we asked whether the migration rates of individual fibroblast strains from a cohort of CLP patients were distributed normally, or whether they clustered into statistically distinct groups. The CLP samples were age-matched since the lip tissue used for isolating the cells was obtained as a by-product of the standard surgery to close the cleft in three months old infants; no biopsy procedure was required. Lip tissue consists of a mucocutaneous zone, i.e. a border between skin epidermis and mucosal epithelium. The same is true for foreskin; thus these two tissues are roughly comparable. Age-matched foreskin fibroblasts therefore appeared to be an acceptable if not an ideal control in our experiments. We are aware that it would be desirable to perform similar experiments with age-matched fibroblasts on the one hand from normal lip tissue, and on the other hand from pure oral mucosa [39]. However, for ethical reasons it is impossible to obtain such tissues from healthy infants. In any case, our comparison of CLP samples among each other is valid without restrictions because they are all derived from exactly the same location.

The majority of CLP lip fibroblast strains migrated with a moderate speed into scratch wounds applied to cell monolayers (“intermediate” group), whereas about one third of the CLP strains closed the wounds at a significantly higher rate (“fast” group). Expectedly, fibroblasts from “fast” strains exhibited more prominent lamellipodia and many but smaller focal adhesions. Normal foreskin fibroblasts used for control were split between the “intermediate” and a third, “slow” migratory group. Interestingly, the “fast” migratory group also included all three strains from patients operated for phimosis. For individual fibroblast strains, the reported phenotypes were stable over several cell passages, indicating that they were due to cell-autonomous differences in gene expression. We therefore measured the expression of a set of genes associated with both CLP and wound healing, and found that mRNA levels were significantly increased for growth factor *TGFA*, and decreased for *PDGFC*, in the “fast” compared to the other two migratory groups. The expression levels of *EGFR* and *PDGFRB*, receptors for TGF-α and PDGF-CC, respectively, followed the same tendency. Although produced by fibroblasts, the major source of PDGFs is blood, where platelet derived PDGF-BB reaches >10 ng/ml in human serum [40]. It is therefore safe to assume that fibroblasts are exposed to high exogenous PDGF concentrations both in wounds and in culture. In contrast, the concentration of TGF-α in serum is very low (ca. 0.15 ng/ml in human [41]). We therefore speculated that differences in migratory speed between individual CLP fibroblast strains were due to variations in the cell-autonomous production and activation of TGF-α. Supporting this notion, the closure of scratch wounds by “intermediate” migrating CLP fibroblast strains was accelerated by addition of 5 ng/ml TGF-α to the culture medium, whereas only higher concentrations had an effect on the “fast” migrating strains. Conversely, specific EGFR inhibitors and TGF-α neutralizing antibodies reduced the speed of migration most effectively for the “fast” strains.

Incidentally, *TGFA* was the very first gene to be implicated in nonsyndromic CLP in humans [42], [43] and since then, many studies have been conducted to corroborate this notion [29]. A direct association of *TGFA* gene polymorphisms with CLP remained inconclusive, however [29], [44], [45]. Therefore, this growth factor is now considered to be a modifier rather than an effector of the CLP phenotype. This is supported by a recent meta-analysis including 29 studies, which provided a positive association of the *TGFA/TaqI* polymorphism with CLP and suggested for *TGFA/BamHI* polymorphism that persons carrying an A1 allele may have a decreased risk of CLP [46]. However, it is not known yet whether these *TGFA* polymorphisms are associated with gain or loss of function. In the animal model, the secondary palate develops normally in *Tgfa* null mice [47], [48], although *Tgfa* is expressed at the medial edge epithelium of fusing palatal shelves [49], and TGF-α was shown to promote mesenchymal cell migration and ECM synthesis in palatal cultures [50]. On the one hand, mice deficient for the TGF-α receptor gene *Egfr* show an increased incidence of orofacial clefts [51], and on the other hand, excess EGF inhibits the fusion of cultured mouse palatal shelves [52]. Thus, both too little and too much EGFR signaling appear to disturb palate morphogenesis. Besides controlling epithelial growth and differentiation, EGFR signaling regulates the expression of matrix metalloproteinases (*MMP*s) [51], [53], [54], which are implicated in palatal morphogenesis as well as wound healing [55]–[60].

A causal relationship between CLP and altered wound healing is best documented for mutations in transcription factor *IRF6*. Homozygous *IRF6^R84C/R84C^* mutant mice develop epithelial fusions between the palatal shelves and the tongue, resulting in cleft palate [61]. In humans, mutations of this gene cause Van der Woude (VWS) syndrome, an orofacial clefting disorder [62]. Importantly, children with VWS were reported to have an increased risk for wound complications following cleft repair [24]. 47% of VWS patients developed wound healing problems, whereas only 19% of non-VWS children were affected. Interestingly, genetic interactions between *TGFA* and *IRF6* (i.e. co-transmission of risk alleles with higher than expected frequency) were reported to contribute to the risk for tooth agenesis as well as orofacial clefts [63]. Similar interactions might occur between *IRF6* and *TGFA* during regenerative processes, and thus changes in TGF-α expression and activity might contribute to altered wound healing in a fraction of CLP patients.

Information on the role of TGF-α in wound healing is relatively sparse. Similar to *Egfr^−/−^* mice, *Tgfa^−/−^* animals have wavy hair and whiskers, pointing to an important function of the TGF-α/EGFR pair in their differentiation [47], [48]. Moreover, *Tgfa^−/−^* mice have underdeveloped eyelids [47] and deficits in wound epithelialization [64]. Perhaps more interesting is the fact that in mice overexpressing TGF-α, hyperplasia of both epithelia and fibroblasts, as well as metaplasia was observed in several organs [65] (reviewed in [23]). Also in humans, dysregulation of Tgf-α is associated with hyperproliferative disease and cancer. Patients with Ménétrier's disease, a hyperproliferative disorder of the stomach, exhibit increased immunoreactivity for TGF-α in their gastric mucosa [66], and mice overexpressing TGF-α recapitulate all symptoms of this disease [65]. Moreover, TGF-α dysregulation has been shown to be causally involved in carcinogenesis of the gastrointestinal tract (reviewed in [67]). In terms of wound regeneration, it is important to note that the administration of EGFR inhibitors (which block EGF as well as TGF-α signaling) for cancer treatment causes adverse cutaneous side effects, and in some cases interferes with wound healing in humans [68], [69]. Taken together, the mentioned studies point to a function of TGF-α not only in morphogenesis of the secondary palate, but also in wound healing and hyperproliferative disorders.

Our finding that CLP patients can be divided into two groups based on migratory behavior of their fibroblasts *in vitro* appears to be based on differential expression and activity of TGF-α. Since the CLP patients investigated in our study are still infants, we do not know yet whether our current *in vitro* results will indeed correlate with differences between the two groups in their wound healing behavior *in vivo*. It will be interesting to follow these patients during the further course of their therapy. In more general terms, our present findings point to an important function of TGF-α in the migration of fibroblasts at the wound edge.

## Supporting Information

Figure S1 **Fluorescence staining of migrating CLP fibroblasts for vinculin and F-actin.** Cultures were fixed 24 h after scratch wounds were applied, and stained with anti-vinculin antibody (green) and phalloidin (red), respectively. Images show a “fast” and an “intermediate” fibroblast strain at low magnification; inserts correspond to the details depicted in Fig. 2. *Scale bar, 50 µm.*
(PPT)

Figure S2 **Relative wound closure rates (RWC) of three individual oral mucosal fibroblast strains.** Cells were isolated from palatal tissue grafts obtained during gingiva recession coverage of 3 healthy adults (Probands 1–3). *In vitro* scratch wound assays were performed to examine whether adult mucosal fibroblasts fell into either the “fast”, “intermediate” or “slow” group. The box plots indicate the median RWC of three independent measurements. Fibroblasts from the second and third passage were used for these experiments. SK was chosen randomly as reference strain of the “intermediate” CLP group. Among the mucosal fibroblasts, no significant difference was measured in terms of the RWCs, which ranged from 25.9% to 32.5%. The RWCs of Probands 2 (25.9%) and 3 (29.9%) were significantly below the one of SK (*p<0.05). Since mucosal fibroblasts are statistically indifferent between each other, and since their RWCs are generally lower than that of the “intermediate” reference strain, on can categorize them as part of the “slow” migratory group.
(PPTX)

Figure S3 **Cell proliferation rate is statistically indifferent between the “fast” and “intermediate” CLP migratory groups.** BrdU incorporation experiments were performed with three randomly chosen CLP fibroblast strains per group (see Materials and Methods). The graph indicates the number of BrdU positive fibroblasts relative to the total number of cells counted after 4 hours of labeling. No differences were measured between “fast” and “intermediate” strains, which excludes the contribution of cell proliferation to the different rates of wound closure *in vitro* by the two groups.
(PPTX)

Table S1 **Primers for qRT-PCR.**
(DOC)

Table S2 **Summary of wound healing assays** ***in vitro*** **.** Scratch wound assays were performed with primary dermal fibroblasts isolated from 16 CLP patients, and with foreskin fibroblasts isolated from 6 healthy individuals (Fsk) and 3 patients with phimosis (Phim). For each cell strain, the table indicates the subject's initials, the subject group (CLP, Fsk, Phim), the median as well as the mean percentage of relative wound closure after 24 hours (% RWC), and its standard error of the mean (SEM; n = 38 single measurements per strain). The entire data set was evaluated by Kruskal-Wallis test followed by a pairwise Wilcoxon rank sum test with Benjamini & Yekutieli (2001) correction for multiple comparisons, which divided the cell strains into 3 statistically distinct migratory groups based on their relative wound closure ability, namely “fast”, “intermediate” and “slow”.
(DOC)

Table S3 **Pairwise Wilcoxon rank sum test with Benjamini & Yekutieli** [32] **correction; data from all strains.** Shown are the p-values for differences in relative wound closure (RWC) from pairwise comparisons of all fibroblast strains used in this study (CLP, cleft lip/palate; Phim, Phimosis; Fsk, normal foreskin). The initials of individual cell donors are indicated at left and on top of the table. Note that fibroblast strains fall into three distinct groups (red = fast, green = intermediate, blue = slow). The p-values for RWC are not significantly different between strains within each group, but for strains from different groups.
(PPT)

Table S4 **Pairwise Wilcoxon rank sum test with Benjamini & Yekutieli** [32] **correction; CLP strains only.** Shown are the p-values for differences in relative wound closure (RWC) from pairwise comparisons of the CLP fibroblast strains used in this study (outlier strain BA was excluded from analysis). The initials of individual cell donors are indicated at left and on top of the table. CLP fibroblast strains fall into two distinct groups (red = fast, green = intermediate). The p-values for RWC are not significantly different between strains within each group, but for strains from different groups.
(PPT)

Movie S1 **Life imaging of “fast” migratory strain XB during 24 hours.**
(MP4)

Movie S2 **Life imaging of “intermediate” migratory strain LP during 24 hours.**
(MP4)
