# Supplementary figures and images for: Accelerated Wound Closure In Vitro by Fibroblasts from a Subgroup of Cleft Lip/Palate Patients: Role of Transforming Growth Factor-α
Source: PLoS One. 2014 Oct 31;9(10):e111752. doi: 10.1371/journal.pone.0111752 (PMC4216129; doi:10.1371/journal.pone.0111752)

## Slide 1
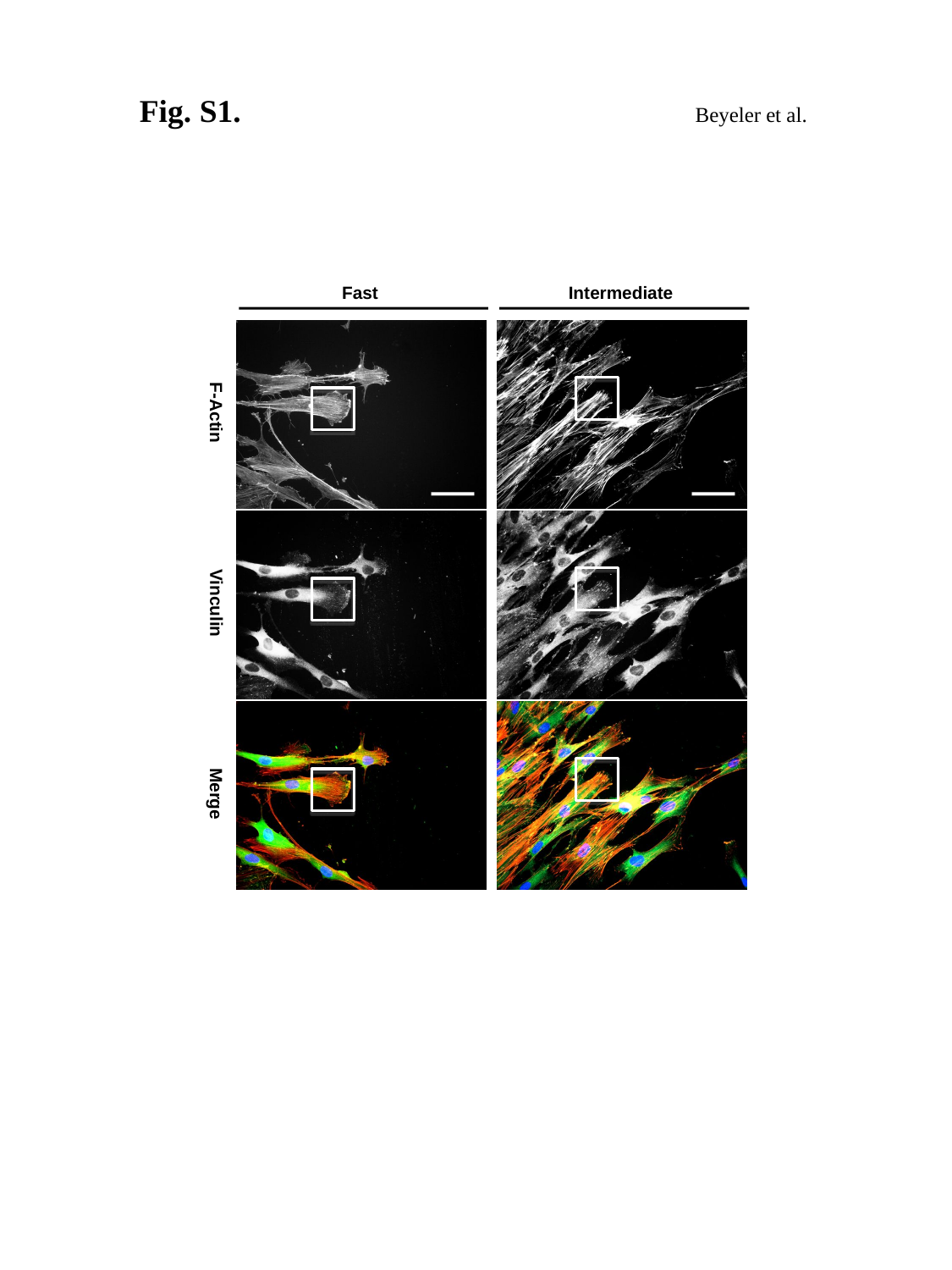

Fig. S1.				 Beyeler et al.
Fast
Intermediate
F-Actin
Vinculin
Merge

Supplement: Figure S1 — Fluorescence staining of migrating CLP fibroblasts for vinculin and F-actin. Cultures were fixed 24 h after scratch wounds were applied, and stained with anti-vinculin antibody (green) and phalloidin (red), respectively. Images show a “fast” and an “intermediate” fibroblast strain at low magnification; inserts correspond to the details depicted in Fig. 2. Scale bar, 50 µm. (PPT) [file pone.0111752.s001.ppt]

## Slide 1
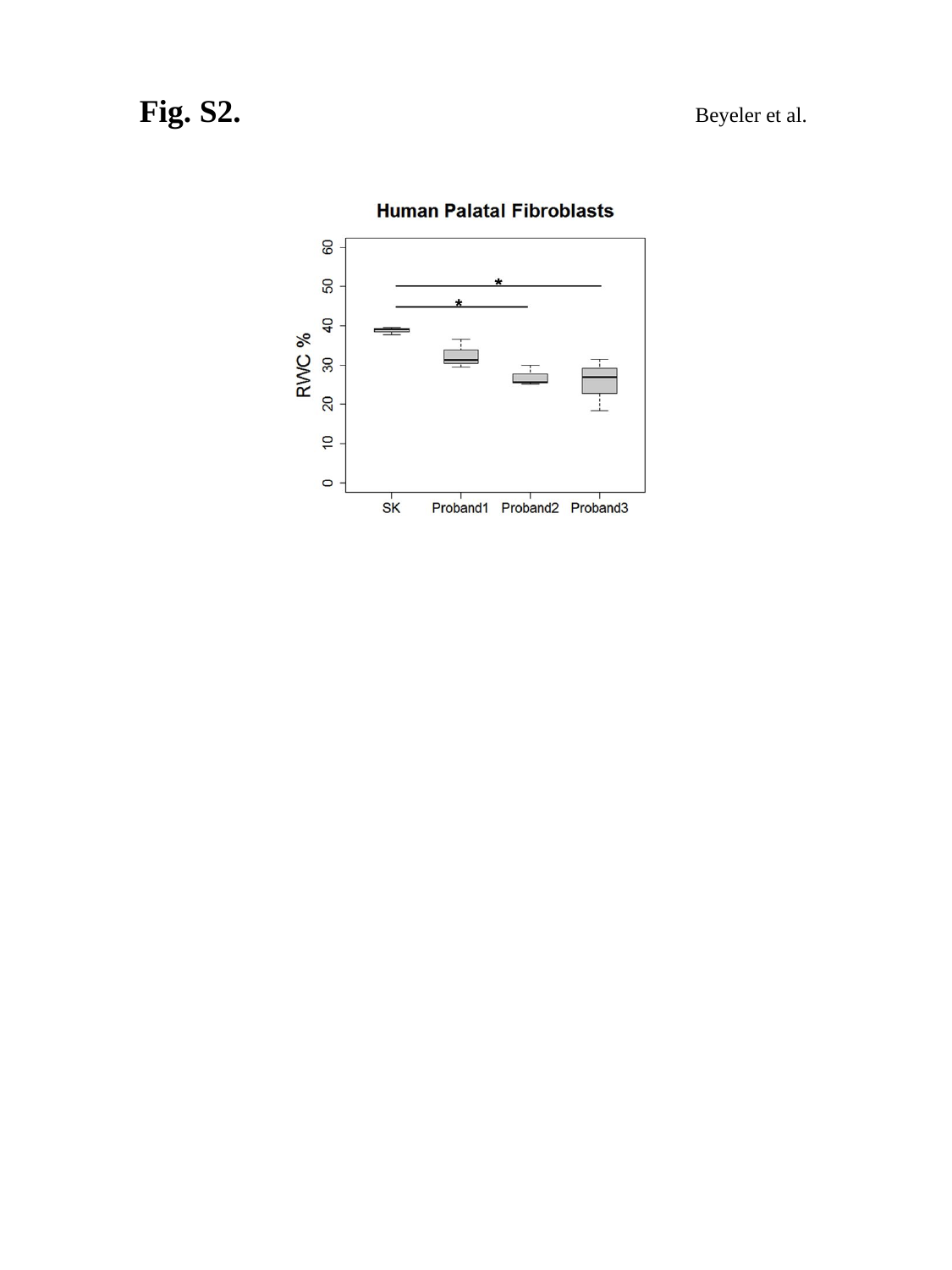

Fig. S2.				 Beyeler et al.

Supplement: Figure S2 — Relative wound closure rates (RWC) of three individual oral mucosal fibroblast strains. Cells were isolated from palatal tissue grafts obtained during gingiva recession coverage of 3 healthy adults (Probands 1–3). In vitro scratch wound assays were performed to examine whether adult mucosal fibroblasts fell into either the “fast”, “intermediate” or “slow” group. The box plots indicate the median RWC of three independent measurements. Fibroblasts from the second and third passage were used for these experiments. SK was chosen randomly as reference strain of the “intermediate” CLP group. Among the mucosal fibroblasts, no significant difference was measured in terms of the RWCs, which ranged from 25.9% to 32.5%. The RWCs of Probands 2 (25.9%) and 3 (29.9%) were significantly below the one of SK (*p<0.05). Since mucosal fibroblasts are statistically indifferent between each other, and since their RWCs are generally lower than that of the “intermediate” reference strain, on can categorize them as part of the “slow” migratory group. (PPTX) [file pone.0111752.s002.pptx]

## Slide 1
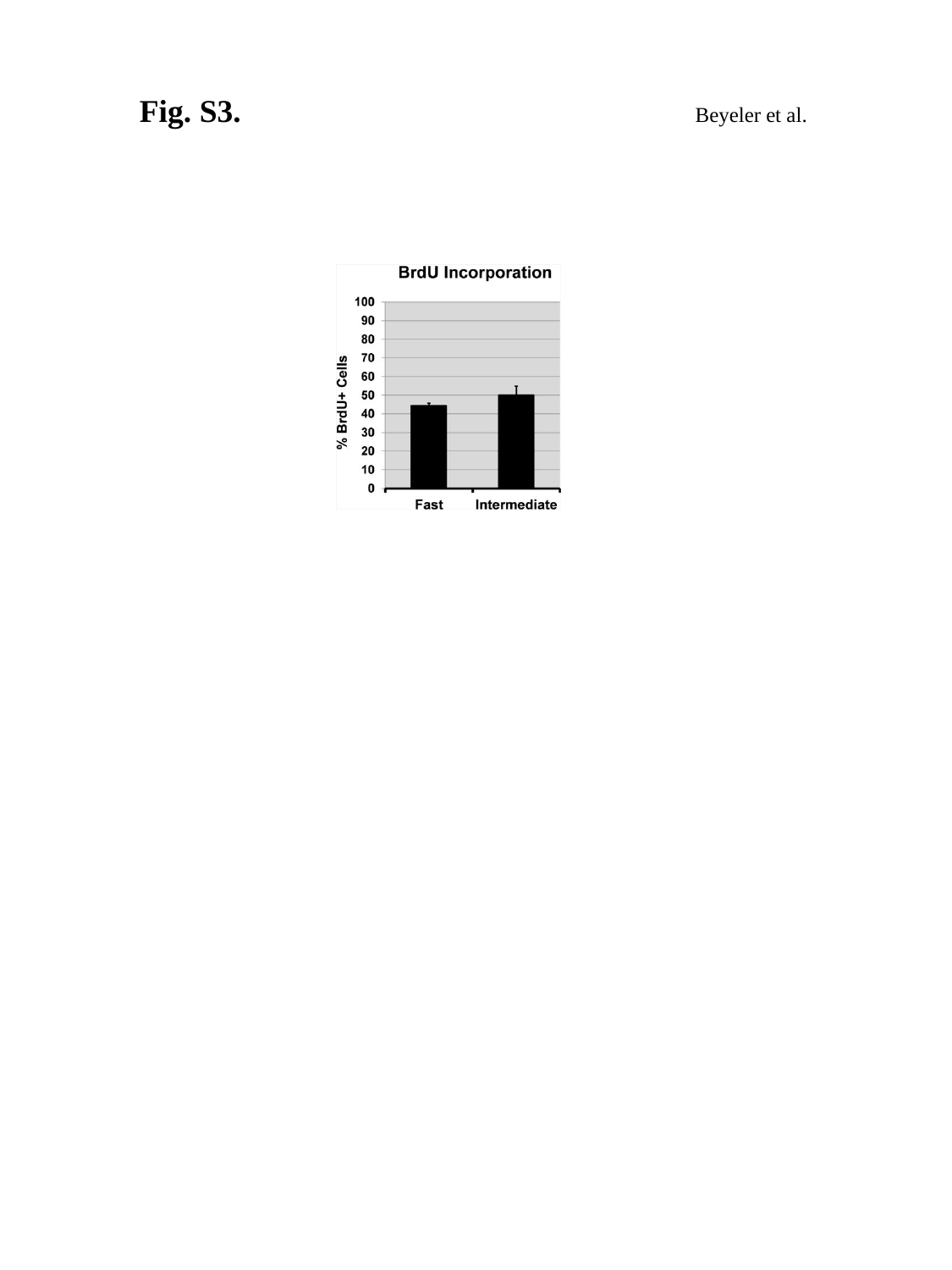

Fig. S3.				 Beyeler et al.

Supplement: Figure S3 — Cell proliferation rate is statistically indifferent between the “fast” and “intermediate” CLP migratory groups. BrdU incorporation experiments were performed with three randomly chosen CLP fibroblast strains per group (see Materials and Methods). The graph indicates the number of BrdU positive fibroblasts relative to the total number of cells counted after 4 hours of labeling. No differences were measured between “fast” and “intermediate” strains, which excludes the contribution of cell proliferation to the different rates of wound closure in vitro by the two groups. (PPTX) [file pone.0111752.s003.pptx]

## Slide 1
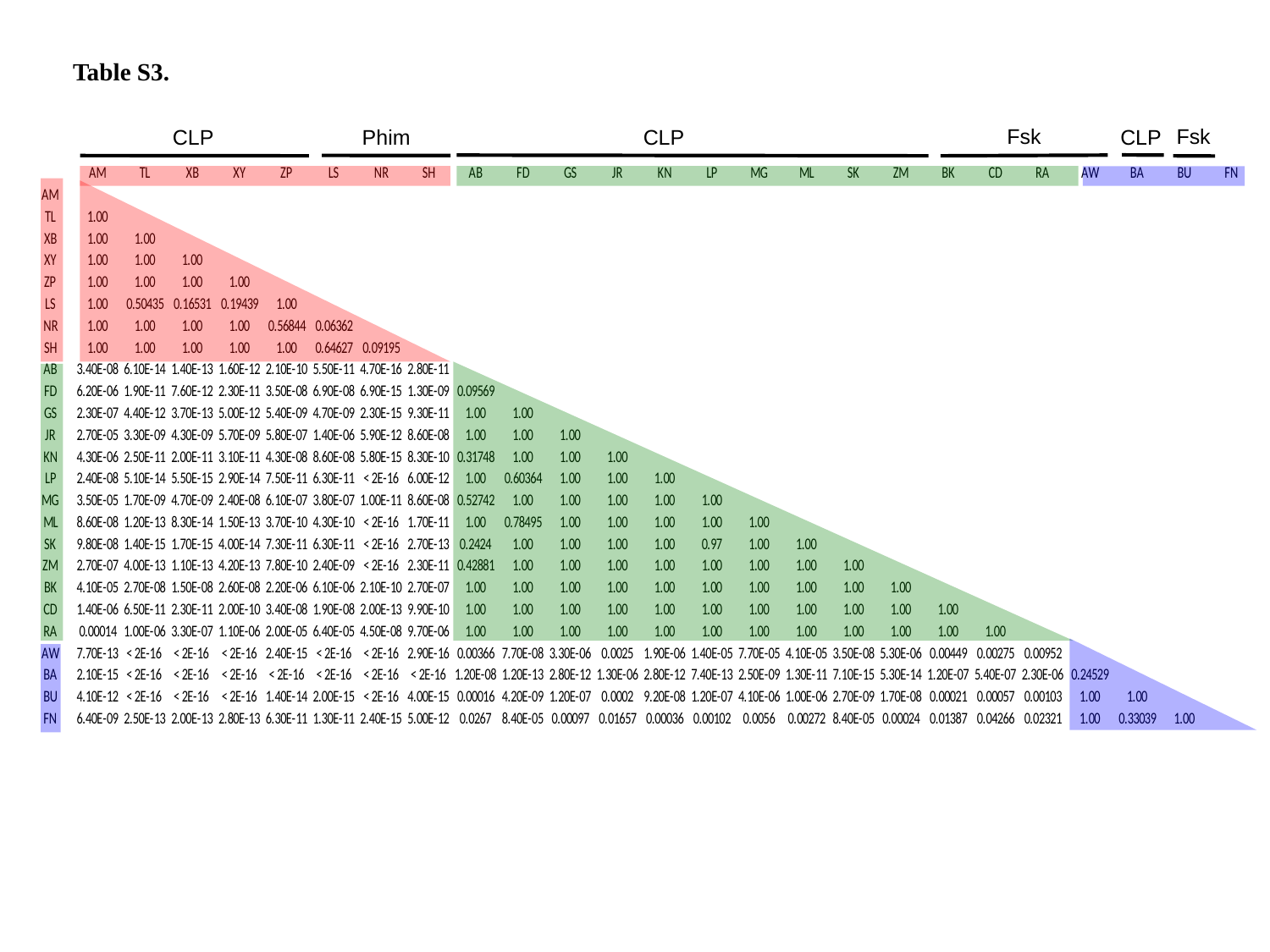

Table S3.
Fsk
Fsk
Phim
CLP
CLP
CLP

Supplement: Table S3 — Pairwise Wilcoxon rank sum test with Benjamini & Yekutieli [32] correction; data from all strains. Shown are the p-values for differences in relative wound closure (RWC) from pairwise comparisons of all fibroblast strains used in this study (CLP, cleft lip/palate; Phim, Phimosis; Fsk, normal foreskin). The initials of individual cell donors are indicated at left and on top of the table. Note that fibroblast strains fall into three distinct groups (red = fast, green = intermediate, blue = slow). The p-values for RWC are not significantly different between strains within each group, but for strains from different groups. (PPT) [file pone.0111752.s006.ppt]

## Slide 1
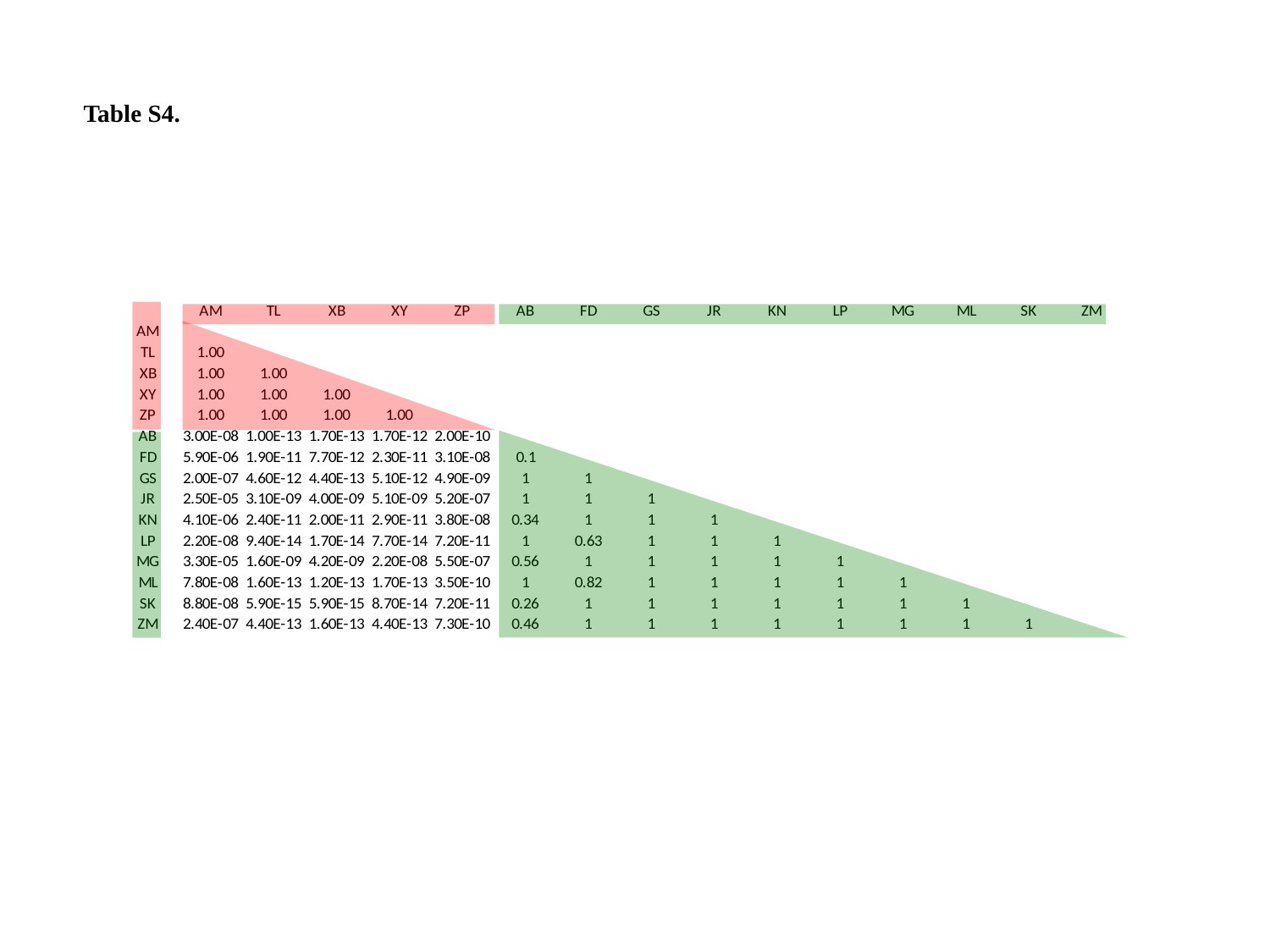

Table S4.

Supplement: Table S4 — Pairwise Wilcoxon rank sum test with Benjamini & Yekutieli [32] correction; CLP strains only. Shown are the p-values for differences in relative wound closure (RWC) from pairwise comparisons of the CLP fibroblast strains used in this study (outlier strain BA was excluded from analysis). The initials of individual cell donors are indicated at left and on top of the table. CLP fibroblast strains fall into two distinct groups (red = fast, green = intermediate). The p-values for RWC are not significantly different between strains within each group, but for strains from different groups. (PPT) [file pone.0111752.s007.ppt]
