# Supplementary material for: Accelerated Wound Closure In Vitro by Fibroblasts from a Subgroup of Cleft Lip/Palate Patients: Role of Transforming Growth Factor-α
Source: PLoS One. 2014 Oct 31;9(10):e111752. doi: 10.1371/journal.pone.0111752 (PMC4216129; doi:10.1371/journal.pone.0111752)
Supplement: Table S1 — Primers for qRT-PCR. (DOC) [file pone.0111752.s004.doc]

**Table S1.** Beyeler et al.

| **Gene** | **Sequence** | | **bp** | **Accession number** |
| --- | --- | --- | --- | --- |
| Transforming growth factor, beta 1 (*TGFB1*) | Forward  Reverse | 5’ TCCTGTGACAGCAGGGATAAC 3’  5’ TCCGTGGAGCTGAAGCAATA 3’ | 200 | [NM_000660.4](http://www.ncbi.nlm.nih.gov/entrez/viewer.fcgi?db=nucleotide&id=260655621) |
| Transforming growth factor, beta 3 (*TGFB3*) | Forward  Reverse | 5’ TACTGCTTCCGCAACTTGGA 3’  5’ GCTTCAGGGTTCAGAGTGTTG 3’ | 200 | [NM_003239.2](http://www.ncbi.nlm.nih.gov/entrez/viewer.fcgi?db=nucleotide&id=169790812) |
| Bone morphogenetic protein 7 (*BMP7*) | Forward  Reverse | 5’ ACAACTCGGCACCCATGTTCA 3’  5’ ATGTTCCACGAGGTTGACGAA 3’ | 200 | [NM_001719.2](http://www.ncbi.nlm.nih.gov/entrez/viewer.fcgi?db=nucleotide&id=187608319) |
| Fibroblast growth factor 12 (*FGF12*) | Forward  Reverse | 5’ ATAGCCAGCTCCTTGATCCG 3’  5’ TTGAGCTGGGGTTCTGGTCT 3’ | 200 | [NM_021032.4](http://www.ncbi.nlm.nih.gov/entrez/viewer.fcgi?db=nucleotide&id=315113875) |
| Epidermal growth factor (*EGF*) | Forward  Reverse | 5’ TGTCCCTTTTTGGTGACCGT 3’  5’ GCAAAGTTTCTGCTCAGGCT 3’ | 200 | [NM_001963.4](http://www.ncbi.nlm.nih.gov/entrez/viewer.fcgi?db=nucleotide&id=296011011) |
| Transforming growth factor, alpha (*TGFA*) | Forward  Reverse | 5’ GTCTGCGTCTTTTTCCCCCG 3’  5’ GGGTCTGCACTCAGCGG 3’ | 200 | [NM_003236.3](http://www.ncbi.nlm.nih.gov/entrez/viewer.fcgi?db=nucleotide&id=345842399) |
| Platelet derived growth factor C (*PDGFC*) | Forward  Reverse | 5’ TTATACAGCTGCACACCTCG 3’  5’ GGTCTCAACTGAAGGACCTCG 3’ | 200 | [NM_016205.2](http://www.ncbi.nlm.nih.gov/entrez/viewer.fcgi?db=nucleotide&id=307691204) |
| Transforming growth factor, beta receptor 2 (*TGFBR2*) | Forward  Reverse | 5’ AGAAGTCGGATGTGGAAATGGA 3’  5’ GTTGCTCATGCAGGATTTCTGG 3’ | 200 | [NM_001024847.2](http://www.ncbi.nlm.nih.gov/entrez/viewer.fcgi?db=nucleotide&id=260655621) |
| Fibroblast growth factor receptor 1 (*FGFR1*) | Forward  Reverse | 5’ CGTATGCCCGTAGCTCCATA 3’  5’ CTCCAGGTGGCATAACGGAC 3’ | 200 | [NM_023110.2](http://www.ncbi.nlm.nih.gov/entrez/viewer.fcgi?db=nucleotide&id=105990521) |
| Epidermal growth factor receptor (*EGFR*) | Forward  Reverse | 5’ ACAACTGTATCCAGTGTGCCC 3’  5’ GATCTTAGGCCCATTCGTTGGA 3’ | 200 | [NM_005228.3](http://www.ncbi.nlm.nih.gov/entrez/viewer.fcgi?db=nucleotide&id=41327737) |
| Platelet-derived growth factor recep-tor, beta (*PDGFRB*) | Forward  Reverse | 5’ ATGCTTAAATCCACAGCCCG 3’  5’ AGGAAGGTGTGTTTGTTGCG 3’ | 200 | [NM_002609.3](http://www.ncbi.nlm.nih.gov/entrez/viewer.fcgi?db=nucleotide&id=68216043) |
| Met proto-oncogene (*MET*) | Forward  Reverse | 5’ GCTTCATGCAGGTTGTGGTT 3’  5’ ACTGCAGGACTGGAAATGTCT 3’ | 200 | [NM_001127500.1](http://www.ncbi.nlm.nih.gov/entrez/viewer.fcgi?db=nucleotide&id=188595715) |
| Jagged 1 (*JAG1*) | Forward  Reverse | 5’ CGCGACGAGTGTGACACATA 3’  5’ AACGTATAGGACCTCGGCCA 3’ | 200 | [NM_000214.2](http://www.ncbi.nlm.nih.gov/entrez/viewer.fcgi?db=nucleotide&id=168480146) |
| Tenascin C (*TNC*) | Forward  Reverse | 5’ TCAAAGACGTGCCAGGAGAC 3’  5’ TCTGTCTGGGAAACACGTCG 3’ | 202 | [NM_002160.3](http://www.ncbi.nlm.nih.gov/entrez/viewer.fcgi?db=nucleotide&id=340745336) |

Table S1. continued Beyeler et al.

| Tenascin W (*TNN*) | Forward  Reverse | 5’ GTGGCTGGATTGTCTTCCAGA 3’  5’ AGCATAGGCAGATTCATTGGC 3’ | 200 | [NM_022093.1](http://www.ncbi.nlm.nih.gov/entrez/viewer.fcgi?db=nucleotide&id=62988323) |
| --- | --- | --- | --- | --- |
| Fibronectin 1 (*FN1*) | Forward  Reverse | 5’ CCATTATTGGGTACCGCATCACA 3’  5’ AGGAGGAACAGCCGTTTGTTGT 3’ | 200 | [NM_212482.1](http://www.ncbi.nlm.nih.gov/entrez/viewer.fcgi?db=nucleotide&id=47132556) |
| Collagen, type I, alpha1 (*COL1A1*) | Forward  Reverse | 5’GAGGCACGCGGAGTGTGA3’  5’TGATTGGTGGGATGTCTTCGTC3’ | 200 | NM_000088.3 |
| Collagen, typeIII, alpha1 (*COL3A1*) | Forward  Reverse | 5’GGCATTCCTTCGACTTCTCTC3’  5’TGTGTTTCGTGCAACCATCC3’ | 200 | NM_000090.3 |
| Matrix metallo-peptidase 2 (*MMP2*) | Forward  Reverse | 5’GTTGGCAGTGCAATACCTGA’3  5’GAGGGAAGAAGTTGTAGTTGGC’3 | 200 | NM_004530.4 |
| Matrix metallo-peptidase 9 (*MMP9*) | Forward  Reverse | 5’CTCTATGGTCCTCGCCCTGAA’3  5’GGCACAGTAGTGGCCGTAGA’3 | 200 | NM_004994.2 |
| Vinculin (*VCL*) | Forward  Reverse | 5’ GCTGAGGTGGGTATAGGTGTT 3’  5’ ATGTCATTGCCCTTACTAGACCAC 3’ | 200 | [NM_014000.2](http://www.ncbi.nlm.nih.gov/entrez/viewer.fcgi?db=nucleotide&id=50593530) |
| Actin, alpha 2, smooth muscle *(ACTA2*) | Forward  Reverse | 5’ AAAGCAAGTCCTCCAGCGTT 3’  5’ GCTTCACAGGATTCCCGTCT 3’ | 200 | [NM_001141945.1](http://www.ncbi.nlm.nih.gov/entrez/viewer.fcgi?db=nucleotide&id=213688374) |
| Alcohol dehydrogen-ase 1C, class I gamma polypeptide (*ADH1C*) | Forward  Reverse | 5‘ CTCAAGCAGAGAAGAAATCCACA 3‘  5‘ CCTGCAGCCACCATCTTAATG 3‘ | 200 | [NM_000669.3](http://www.ncbi.nlm.nih.gov/entrez/viewer.fcgi?db=nucleotide&id=71565150) |
| Interferon regulatory factor 6 (*IRF6*) | Forward  Reverse | 5‘ GCTCTTCCATATCATGGCCCTC 3‘  5‘ CTACAGCCCAGGCCTTAAAAA 3‘ | 200 | [NM_006147.3](http://www.ncbi.nlm.nih.gov/entrez/viewer.fcgi?db=nucleotide&id=331999973) |
| Runt-related trans-cription factor 2 (*RUNX2*) | Forward  Reverse | 5‘ TCCACAAGGACAGAGTCAGAT 3‘  5‘ GTTCTGAAGCACCTGAAATGCG 3‘ | 200 | [NM_001015051.3](http://www.ncbi.nlm.nih.gov/entrez/viewer.fcgi?db=nucleotide&id=226442790) |
| SRY (sex determining region Y)-box 9 (*SOX9*) | Forward  Reverse | 5‘ AAGCTCTGGAGACTTCTGAACG 3‘  5‘ AGCGCCTTGAAGATGGCGT 3‘ | 200 | [NM_000346.3](http://www.ncbi.nlm.nih.gov/entrez/viewer.fcgi?db=nucleotide&id=182765453) |
| Glyceraldehyde-3-phosphate dehydro-genase (*GAPDH*) | Forward  Reverse | 5’ CTCTGACTTCAACAGCGACACCC 3’  5’ TCCTCTTGTGCTCTTGCTGGGGC 3’ | 200 | [NM_001256799.1](http://www.ncbi.nlm.nih.gov/entrez/viewer.fcgi?db=nucleotide&id=378404907) |
