# Supplementary material for: Accelerated Wound Closure In Vitro by Fibroblasts from a Subgroup of Cleft Lip/Palate Patients: Role of Transforming Growth Factor-α
Source: PLoS One. 2014 Oct 31;9(10):e111752. doi: 10.1371/journal.pone.0111752 (PMC4216129; doi:10.1371/journal.pone.0111752)
Supplement: Table S2 — Summary of wound healing assays in vitro . Scratch wound assays were performed with primary dermal fibroblasts isolated from 16 CLP patients, and with foreskin fibroblasts isolated from 6 healthy individuals (Fsk) and 3 patients with phimosis (Phim). For each cell strain, the table indicates the subject's initials, the subject group (CLP, Fsk, Phim), the median as well as the mean percentage of relative wound closure after 24 hours (% RWC), and its standard error of the mean (SEM; n = 38 single measurements per strain). The entire data set was evaluated by Kruskal-Wallis test followed by a pairwise Wilcoxon rank sum test with Benjamini & Yekutieli (2001) correction for multiple comparisons, which divided the cell strains into 3 statistically distinct migratory groups based on their relative wound closure ability, namely “fast”, “intermediate” and “slow”. (DOC) [file pone.0111752.s005.doc]

**Table S2. Beyeler et al.**

| **CLP Derived Fibroblasts** | | | | | | | | | | | | | | | | | | | | | |
| --- | --- | --- | --- | --- | --- | --- | --- | --- | --- | --- | --- | --- | --- | --- | --- | --- | --- | --- | --- | --- | --- |
| **Fast** | | | | | | |  | **Intermediate** | | | | | |  | **Slow** | | | | | | |
| **Initials** | **Median** | | Mean | | SEM | |  | **Initials** | **Median** | Mean | | SEM | |  | **Initials** | **Median** | | Mean | | SEM | |
| **AM** | **41.8** | | 43.8 | | 3.9 | |  | **AB** | **33.9** | 33.5 | | 1.2 | |  | **BA** | **26.1** | | 27.6 | | 2.3 | |
| **TL** | **43.1** | | 44.3 | | 2.9 | |  | **FD** | **35.8** | 35.2 | | 1.5 | |  |  |  |  | | |  | |
| **XB** | **46.4** | | 45.0 | | 1.7 | |  | **GS** | **35.0** | 35.6 | | 2.5 | |  |  |  |  | | |  | |
| **XY** | **45.2** | | 44.6 | | 2.2 | |  | **JR** | **34.1** | 34.4 | | 0.9 | |  |  |  |  | | |  | |
| **ZP** | **44.3** | | 44.4 | | 2.5 | |  | **KN** | **35.0** | 35.3 | | 1.9 | |  |  |  |  | | |  | |
|  |  |  | | |  | |  | **LP** | **33.2** | 34.2 | | 1.6 | |  |  |  |  | | |  | |
|  |  |  | | |  | |  | **MG** | **35.5** | 35.2 | | 0.9 | |  |  |  |  | | |  | |
|  |  |  | | |  | |  | **ML** | **35.0** | 34.5 | | 2.4 | |  |  |  |  | | |  | |
|  |  |  | | |  | |  | **SK** | **35.2** | 35.3 | | 2 | |  |  |  |  | | |  | |
|  |  |  | | |  | |  | **ZM** | **35.2** | 35.6 | | 1.6 | |  |  |  |  | | |  | |
| **Range** | **41.8 -**  **46.4%** | | | 43.8 -  45.0% | |  |  |  | **33.2 -**  **35.8%** | | 33.5 - 35.6% | |  |  | **-** | | | | | | |
|  |  |  |  |  |  | | | | | | |
| **Average** | **44.2%** | | | 44.4% | |  |  |  | **34.8 %** | | 34.9% | |  |  | **-** | | | | | | |
|  |  | | | | |  |  |  |  | | | |  |  |  |  | | | | | |
| **Foreskin and Phimosis Derived Fibroblasts Strains** | | | | | | | | | | | | | | | | | | | | | |
| **Fast (Phim)** | | | | | | |  | **Intermediate (Fsk)** | | | | | |  | **Slow (Fsk)** | | | | | | |
| **Initials** | **Median** | | Mean | | SEM | |  | **Initials** | **Median** | Mean | | SEM | |  | **Initials** | **Median** | | Mean | | SEM | |
| **LS** | **42.5** | | 42.8 | | 1.1 | |  | **BK (Fsk)** | **35.0** | 34.7 | | 0.6 | |  | **AW (Fsk)** | **28.7** | | 29.4 | | 1.4 | |
| **NR** | **44.1** | | 45.5 | | 2.0 | |  | **CD (Fsk)** | **34.5** | 34.0 | | 2.5 | |  | **BU (Fsk)** | **28.6** | | 28.4 | | 0.4 | |
| **SH** | **43.8** | | 45.0 | | 3.2 | |  | **RA (Fsk)** | **33.7** | 35.6 | | 2.7 | |  | **FN (Fsk)** | **29.6** | | 29.8 | | 0.6 | |
| **Range** | **42.5 - 44.1%** | | | 42.8 - 45.5% | |  |  |  | **33.7 - 35.0%** | | 34.0 - 35.6% | |  |  |  | **28.6 - 29.6%** | | | 28.4 - 29.8% | |  |
|  |  |  |  |  |  |  |
| **Average** | **44.0%** | | | 44.4% | |  |  |  | **34.4%** | | 34.8% | |  |  |  | **29.0%** | | | 29.2% | |  |
|  | | | | | | | | | | | | | | | | | | | | | |
| **RWC % of Migratory Groups: Summary** | | | | | | | | | | | | | | | | | | | | | |
| **Fast (CLP + Phim)** | | | | | | |  | **Intermediate (CLP + Fsk)** | | | | | |  | **Slow (Fsk + CLP)** | | | | | | |
| **Range** | **41.8 - 46.4%** | | 42.8 - 45.5% | |  | |  |  | **33.2 - 35.8%** | 33.5 - 35.6% | |  | |  |  | **26.1 - 29.6%** | | 27.6 - 29.8% | |  | |
|  | |  |  |  | |  |  |  | |
| **Average** | **44.1%** | | 44.4% | |  | |  |  | **34.7%** | 34.9% | |  | |  |  | **28.2%** | | 28.4% | |  | |
